# Loss of G0/G1 switch gene 2 (G0S2) promotes disease progression and drug resistance in chronic myeloid leukaemia (CML) by disrupting glycerophospholipid metabolism

**DOI:** 10.1002/ctm2.1146

**Published:** 2022-12-19

**Authors:** Mayra A. Gonzalez, Idaly M. Olivas, Alfonso E. Bencomo‐Alvarez, Andres J. Rubio, Christian Barreto‐Vargas, Jose L. Lopez, Sara K. Dang, Jonathan P. Solecki, Emily McCall, Gonzalo Astudillo, Vanessa V. Velazquez, Katherine Schenkel, Kelaiah Reffell, Mariah Perkins, Nhu Nguyen, Jehu N. Apaflo, Efren Alvidrez, James E. Young, Joshua J. Lara, Dongqing Yan, Anna Senina, Jonathan Ahmann, Katherine E. Varley, Clinton C. Mason, Christopher A. Eide, Brian J. Druker, Md Nurunnabi, Osvaldo Padilla, Sudip Bajpeyi, Anna M. Eiring

**Affiliations:** ^1^ Department of Molecular and Translational Medicine Center of Emphasis in Cancer Texas Tech University Health Sciences Center El Paso El Paso Texas USA; ^2^ L. Frederick Francis Graduate School of Biomedical Sciences Texas Tech University Health Sciences Center El Paso El Paso Texas USA; ^3^ Paul L. Foster School of Medicine, Texas Tech University Health Sciences Center El Paso El Paso Texas USA; ^4^ Immunology Division University of Guadalajara Guadalajara Jalisco Mexico; ^5^ Metabolic, Nutrition and Exercise Research (MiNER) Laboratory, Department of Kinesiology University of Texas at El Paso El Paso Texas USA; ^6^ Department of Pharmaceutical Sciences School of Pharmacy University of Texas at El Paso El Paso Texas USA; ^7^ Huntsman Cancer Institute The University of Utah Salt Lake City Utah USA; ^8^ Knight Cancer Institute Division of Hematology/Medical Oncology Oregon Health & Science University Portland Oregon USA; ^9^ Department of Pathology Texas Tech University Health Sciences Center El Paso El Paso Texas USA

**Keywords:** chronic myeloid leukaemia (CML), G0/G1 switch gene 2 (G0S2), glycerophospholipid metabolism, tyrosine kinase inhibitor (TKI) resistance

## Abstract

Tyrosine kinase inhibitors (TKIs) targeting BCR::ABL1 have turned chronic myeloid leukaemia (CML) from a fatal disease into a manageable condition for most patients. Despite improved survival, targeting drug‐resistant leukaemia stem cells (LSCs) remains a challenge for curative CML therapy. Aberrant lipid metabolism can have a large impact on membrane dynamics, cell survival and therapeutic responses in cancer. While ceramide and sphingolipid levels were previously correlated with TKI response in CML, the role of lipid metabolism in TKI resistance is not well understood. We have identified downregulation of a critical regulator of lipid metabolism, G0/G1 switch gene 2 (G0S2), in multiple scenarios of TKI resistance, including (1) BCR::ABL1 kinase‐independent TKI resistance, (2) progression of CML from the chronic to the blast phase of the disease, and (3) in CML versus normal myeloid progenitors. Accordingly, CML patients with low *G0S2* expression levels had a worse overall survival. G0S2 downregulation in CML was not a result of promoter hypermethylation or BCR::ABL1 kinase activity, but was rather due to transcriptional repression by MYC. Using CML cell lines, patient samples and G0s2 knockout (G0s2^−/−^) mice, we demonstrate a tumour suppressor role for G0S2 in CML and TKI resistance. Our data suggest that reduced G0S2 protein expression in CML disrupts glycerophospholipid metabolism, correlating with a block of differentiation that renders CML cells resistant to therapy. Altogether, our data unravel a new role for G0S2 in regulating myeloid differentiation and TKI response in CML, and suggest that restoring G0S2 may have clinical utility.

## Graphical abstract

1. G0S2 is reduced in disease progression and drug resistance of CML.
2. G0S2 expression impairs survival of CML progenitors with little effect on apoptosis.
3. G0S2 function in CML is independent of its known function as an inhibitor of adipose triglyceride lipase (ATGL), the rate‐limiting enzyme for intracellular lipolysis.
4. Loss of G0S2 disrupts glycerophospholipid metabolism in CML and promotes therapy resistance.

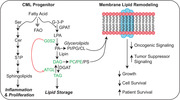

## 1 INTRODUCTION

Chronic myeloid leukaemia (CML) is caused by BCR::ABL1, a constitutively active fusion tyrosine kinase.^1^ A majority of CML patients present in the chronic phase (CP‐CML), where ABL1 tyrosine kinase inhibitors (TKIs) have revolutionised disease therapy, turning it from a lethal disease into a manageable condition for most patients. Despite the success, 10%–15% of patients fail first‐line TKI therapy,^2^ necessitating treatment changes to limit the risk of progression to the rapidly fatal blast phase of CML (BP‐CML), characterised by differentiation blockade and therapy resistance.^3^, ^4^ Additionally, TKIs do not eliminate the residual CML leukaemic stem cell (LSC) population,^5^, ^6^ with disease recurrence common after TKI discontinuation.^7^, ^8^, ^9^ Thus, new therapeutic combination strategies are required to overcome persistent CML LSCs to minimise the risk of progression and improve rates and durability of treatment‐free remission.

Half of clinical TKI resistance is free of BCR::ABL1 kinase domain mutations,^10^ suggesting BCR::ABL1 kinase‐independent resistance mechanisms, the primary form of TKI resistance in CML LSCs.^11^ From a previously reported gene expression classifier study that predicted a patient's response to first‐line imatinib, mRNA encoding G0/G1 switch gene 2 (*G0S2*) was identified among the most downregulated genes in TKI resistance.^12^ G0S2 is a small protein that regulates multiple cellular functions, including apoptosis,^13^ quiescence,^14^, ^15^ lipolysis,^16^, ^17^, ^18^ de novo lipogenesis^19^ and oxidative phosphorylation (OxPhos).^20^, ^21^, ^22^ Additionally, *G0S2* is an all‐trans retinoic acid (ATRA) target gene in acute promyelocytic leukaemia (APL).^18^, ^23^, ^24^ In the K562 CML cell line and normal haematopoietic stem cells (HSCs), G0S2 inhibits proliferation by direct interaction with nucleolin^14^, ^15^; however, its role in CML blastic transformation and TKI resistance remains unknown. We hypothesised that G0S2 plays a tumour suppressor role in CML, and that downregulation of G0S2 contributes to reduced responses to TKI therapy. Altogether, our findings suggest that loss of G0S2 occurs in multiple contexts of TKI resistance and progression in CML, and that restoring G0S2 expression in such scenarios may have clinical utility by promoting myeloid differentiation and restoring TKI sensitivity.

## 2 MATERIALS AND METHODS

### 2.1 Cell lines and patient samples

Details regarding the culture of cell lines and primary cells are available in Supporting Information. Mononuclear cells (MNCs) from cord blood (CB) or peripheral blood (PB) of CML patients (see Table S1) were separated by density centrifugation on Ficoll‐Paque PREMIUM (GE Healthcare Systems, Chicago, IL, USA). CD34^+^ cells were selected using an autoMACS system (Miltenyi Biotec, San Diego, CA, USA) or the EasySep Human CD34 Positive Selection Kit II (Stem Cell Technologies, Vancouver, British Columbia, Canada). Samples were confirmed to have >90% purity by flow cytometry on a BD FACSCanto (BD Biosciences, San Jose, CA, USA). All CML cells were confirmed to harbour native *BCR::ABL1* as previously described (see Table S1).^25^ Patient samples that did not have a *BCR::ABL1* kinase domain mutation were specifically selected for use in this study, and all BP‐CML specimens were confirmed to be exclusively myeloid in nature. Fresh or frozen CD34^+^ cells from CML patients or normal CB were cultured in Iscove's Modified Dulbecco's Medium (IMDM, Life Technologies, Carlsbad, CA, USA) supplemented with 10% BIT9500 (Stem Cell Technologies), 100 U/ml penicillin–streptomycin (Life Technologies), 2 mM L‐glutamine (Life Technologies) and recombinant cytokines (CC100; Stem Cell Technologies) or human granulocyte‐colony stimulating factor (hG‐CSF, 25 ng/ml, 7–10 days) (Peprotech, Inc., Cranbury, NJ, USA). Where indicated, cells were treated with the BCR::ABL1 TKI, imatinib (1 µM, Seleck Chemicals, Houston, TX, USA) or the MYC proto‐oncogene (MYC) inhibitors, MYCi361 or MYCi975 (6 µM, Selleck Chemicals). All patients gave informed consent in accordance with the Declaration of Helsinki, and all studies were approved by the Institutional Review Boards (IRBs) at the University of Utah, Texas Tech University Health Sciences Center El Paso and Oregon Health & Science University.

### 2.2 Reverse transcription‐quantitative polymerase chain reaction

RNA extraction was performed using the RNeasy Mini Kit (Qiagen, Hilden, Germany) and converted to cDNA with the iScript cDNA Synthesis Kit (Bio‐Rad, Hercules, CA, USA). Human *G0S2*, *ATGL*, *BCR::ABL1* and *GUSB* levels were measured by reverse transcription‐quantitative polymerase chain reaction (RT‐qPCR) using the SsoAdvanced SYBR Green Supermix (Bio‐Rad) in a CFX96 Real‐Time PCR Detection System (Bio‐Rad). Murine *G0s2* and *Gapdh* levels were measured using the Luna Universal One‐Step qPCR Kit (New England Biolabs, Ipswich, MA, USA) on a StepOnePlus Real‐Time PCR System (Applied Biosystems, Foster City, CA, USA). Primers are listed in Table S2. Assays were performed in triplicate, and relative expression was analysed using the comparative cycle threshold method (2^−ΔΔCt^).

### 2.3 Immunoblot

CML cell lines and primary CD34^+^ cells were cultured under the indicated conditions (24–72 h). Following drug or cytokine exposure, cells were lysed (4°C; 30 min) in Radio‐Immunoprecipitation Assay (RIPA) buffer (Cell Signaling Technology, Danvers, MA, USA) containing protease (Complete Mini, Roche, Basel, Switzerland) and phosphatase (PhosStop, Roche) inhibitors. Samples were denatured (100°C; 10 min) prior to SDS‐PAGE and transferred to polyvinylidene difluoride (PVDF) membranes. Antibodies are listed in Table S3. Densitometry was conducted using ImageJ (National Institutes of Health, Bethesda, MD, USA).

### 2.4 Plasmids, virus packaging and infection

shRNAs targeting human *G0S2* (shG0S2), murine *G0s2* (shG0s2) or human *ATGL* (shATGL) were purchased from Cellecta (Mountain View, CA, USA, Table S4). Constructs contain the wild‐type tetracycline repressor, and vector expression requires culture with 0.1 µg/ml doxycycline (72 h). For ectopic expression, *G0S2* was PCR amplified from MNCs of a healthy donor and subcloned into the indicated vectors (see Supporting Information). pCDH‐puro‐cMyc was a gift from Jialiang Wang (Addgene #46970).^26^ MSCV‐BCR::ABL1‐IRES‐GFP was a kind gift from Michael Deininger.^27^, ^28^ Lentivirus‐producing 293FT cells (Thermo Fisher Scientific, Waltham, MA, USA) were cultured in Dulbecco's Modified Eagle Medium (DMEM, Life Technologies) plus 10% fetal bovine serum (FBS, Thermo Fisher Scientific), 2.0 mM L‐glutamine (Thermo Fisher Scientific), 1.0 mM sodium pyruvate (Life Technologies), 0.1 mM Minimum Essential Medium (MEM) non‐essential amino acids (Life Technologies) and 100 U/ml penicillin–streptomycin (Life Technologies). Retrovirus‐producing 293T/17 cells were purchased from American Type Culture Collection (Manassas, VA, USA) and cultured in 10% FBS, 2.0 mM L‐glutamine and 100 U/ml penicillin– streptomycin. Lentiviral constructs were packaged in combination with vesicular stomatitis virus glycoprotein (VSV‐G) (Clontech Laboratories, Inc., Mountain View, CA, USA) and psPax2 (Cellecta), and concentrated 100× using polyethylene glycol 8000 (Thermo Fisher Scientific). Retrovirus constructs were packaged in combination with VSV‐G and pCL‐Eco (Addgene), and crude retroviral supernatants were used for transduction of cells in vitro. Viral vectors are listed in Table S4. We transfected virus‐producing cells using the ProFection Mammalian Transfection System (Promega, Madison, WI, USA). Derivative lines were generated by spinoculation of viral particles into cells, followed by fluorescence‐activated cell sorting (FACS) of green fluorescent protein‐positive (GFP^+^) cells or selection in puromycin (2 µg/ml, 72 h).

### 2.5 Clonogenic assays

Methylcellulose clonogenic assays were performed by plating cells (10^3^) in 0.9% MethoCult (Stem Cell Technologies). Primary CD34^+^ cells were cultured with cytokines (CC100, Stem Cell Technologies) in the presence or absence of 1 µM imatinib and/or the indicated cytokines added directly to the medium. Cells were incubated in humid chambers at 37°C ± 0.1 µg/ml doxycycline in duplicate. Colonies were scored after 1–2 weeks in culture.

### 2.6 Flow cytometry

To detect apoptosis, APC‐AnnexinV (BD Biosciences) was used with 7‐aminoactinomycin D (eBioscience, San Diego, CA, USA). To assess *G0S2* expression in stem versus progenitor cells, CD34^+^ cells from CB or CP‐CML patients were sorted by FACS for HSCs, multipotent progenitors (MPPs), common myeloid progenitors (CMPs), granulocyte–macrophage progenitors (GMPs) and megakaryocyte–erythrocyte progenitors (MEPs)^29^ (see Supporting Information and Table S3).

### 2.7 DNA bisulphite conversion and patch PCR sequencing

DNA bisulphite conversion and patch PCR sequencing were performed on DNA from CD34^+^ cells from normal CB or CP‐CML, BP‐CML or TKI‐resistant CML patients.^30^ Sequencing reads were aligned to the reference genome (hg19) using Bismark software.^31^ For additional details, see Supporting Information.

### 2.8 Chromatin immunoprecipitation

TKI‐sensitive K562^S^ or TKI‐resistant K562^R^ cells (2 × 10^6^) were crosslinked using 18% formaldehyde for 10 min at 37°C followed by quenching with 1.25 M glycine for 5 min at room temperature. Nuclear extracts were subjected to chromatin immunoprecipitation (ChIP) as outlined in Supporting Information.

### 2.9 Animal models

Lineage‐negative bone marrow (BM) cells from 6‐week‐old wild‐type or G0s2^−/−^ mice^32^ were selected using the EasySep Mouse Hematopoietic Progenitor Cell Isolation Kit (Stem Cell Technologies). Resulting lineage‐negative cells were cultured in recombinant murine kit ligand (10 ng/ml), interleukin (IL)‐3 (2 ng/ml), IL‐6 (1.2 ng/ml), Flt3 ligand (5 ng/ml) and granulocyte‐macrophage‐colony stimulating factor (GM‐CSF, 5 ng/ml) (Peprotech), and either cultured ± murine G‐CSF (mG‐CSF, 25 ng/ml, 7–10 days) in in vitro differentiation assays or transduced with the MSCV‐BCR::ABL1‐IRES‐GFP vector. In the latter experiment, 3 × 10^5^ unsorted GFP^−/+^ cells were injected intravenously into lethally irradiated (2 × 450 Rad, RS 2000, Rad Source Technologies, Inc., Buford, GA, USA) recipient mice. *BCR::ABL1* expression was confirmed in the peripheral blood of recipient mice by RT‐qPCR. GFP^+^ cells in the BM of moribund recipient mice were quantified by flow cytometry on a BD FACSCanto (BD Biosciences). Haematoxylin and eosin staining of recipient spleens (4 weeks post‐injection, *n* = 3 mice/group) was performed at the University of Texas Health Sciences Center San Antonio Department of Pathology and Laboratory Medicine (San Antonio, TX, USA). Complete blood counts were obtained using a HemaVet 950 haematology analyser (Drew Scientific, Miami Lakes, FL, USA). All experiments were approved by the Institutional Animal Care and Use Committee at Texas Tech University Health Sciences Center El Paso. See Supporting Information for details on subcutaneous injections.

### 2.10 Gene expression analyses

Cell line RNA sequencing (RNAseq) is described in Supporting Information (GSE171945). Gene‐level expression data for *G0S2* were obtained from primary CML patient MNCs isolated from either peripheral blood or BM and subjected to paired‐end 2×150 bp RNAseq using the Illumina HiSeq platform.^33^ Samples were obtained following informed consent in association with Oregon Health & Science University IRB protocol #4422. Patient samples were separated by disease phase or TKI response: CP‐CML (*n* = 53), accelerated phase CML (AP‐CML, *n* = 12), BP‐CML (*n* = 13), newly diagnosed CP‐CML (*n* = 21) and TKI‐resistant CML (*n* = 42). All TKI‐resistant CML patient samples had BCR::ABL1 kinase‐independent resistance, defined by loss of a molecular and/or cytogenetic response to one or more TKIs without the presence of an explanatory BCR::ABL1 kinase domain mutation.^33^ Gene expression analyses using publicly available data are described in Supporting Information.

### 2.11 Lipid profiling

Cells were cultured ± doxycycline (0.1 µg/ml, 72 h) to induce G0S2 ectopic expression or knockdown. Altered G0S2 expression was confirmed at the mRNA and protein levels prior to analyses. Liquid chromatography (LC)/mass spectrometry (MS)‐based lipidomics experiments were performed at Creative Proteomics (Shirley, NY, USA). For LC/MS‐based lipidomics analyses, lipids were isolated using chloroform:MeOH (2:1) and centrifuged for 10 min at 3000 rpm at 4°C. The lower phase was transferred to a new tube for evaporation and dried under liquid nitrogen. Dried extracts were suspended in 200 µl isopropanol:MeOH (1:1) for internal standard analysis. Separation was performed by ultra‐performance liquid chromatography (Thermo, Ultimate 3000LC). The LC system was comprised of a Phenomenex Kinetex C18 (100 mm × 2.1 mm, 1.7 µm) column. The flow rate of the mobile phase was 0.3 ml/min. The column temperature was maintained at 40°C, and the sample manager temperature was set at 4°C. The raw data were acquired and aligned using the LipidSearch^TM^ software (Thermo) based on the *m*/*z* value and the retention time of the ion signals. Ions from either ESI– or ESI+ were merged and imported into the SIMCA‐P software program version v.14.1 (Umetrics, Umea, Sweden) for multivariate analysis. A principal components analysis (PCA) was first used as an unsupervised method for data visualisation and outlier identification. Supervised regression modelling was then performed on the dataset by use of partial least squares discriminant analysis (PLS‐DA) or orthogonal partial least squares discriminant analysis (OPLS‐DA) to identify the potential lipid biomarkers. The biomarkers were filtered and confirmed by combining the results of the VIP values (VIP > 1.5) with a fold change (FC) of >2. To investigate the latent relationships of the lipids, we constructed a correlation network diagram based on Kyoto Encyclopedia of Genes and Genomes (KEGG) databases. All significant lipids were imported to obtain the categorical lipid annotations. Pathway enrichment analysis of the lipids dysregulated by G0S2 ectopic expression or knockdown was performed using Lipid Pathway Enrichment Analysis (https://lipea.biotec.tu‐dresden.de/home, Biomedical Cybernetics Group, Dresden, Germany).

### 2.12 Statistical analyses

All experiments were performed in triplicate unless otherwise noted. Correlation of *G0S2* mRNA levels with survival in CML was established using survival data available for 35 patients from the McWeeney et al. microarray.^12^ CEL files from the original study^12^ were imported with Partek software (Partek Inc., St. Louis, MO, USA) followed by GeneChip‐Robust Multiarray Averaging (GC‐RMA) normalisation. Expression levels were calculated from the microarray (HG‐U133A, Affymetrix, Inc., Santa Clara, CA, USA) and dichotomised into high and low groups based on data distribution. Overall survival was assessed with Kaplan–Meier curves generated in Prism v6.04 (GraphPad Software Inc., San Diego, CA, USA). Statistical analyses were performed in SAS v9.3 (SAS Institute, Cary, NC, USA), and *p‐*values are two‐sided from a log‐rank test. A two‐tailed Student's *t*‐test was used for cell line, mouse and patient sample data demonstrating equivocal variance; the Wilcoxon–Mann–Whitney test was used for unequivocal data.^34^ Paired data were assessed with the Wilcoxon signed‐rank test. Statistical analyses were performed using Microsoft Excel 2013 (Redmond, WA, USA) or Prism v7 (GraphPad Software Inc.).

## 3 RESULTS

### 3.1 G0S2 is downregulated in CML disease progression and imatinib resistance in a BCR::ABL1 kinase‐independent manner

**FIGURE 1:**
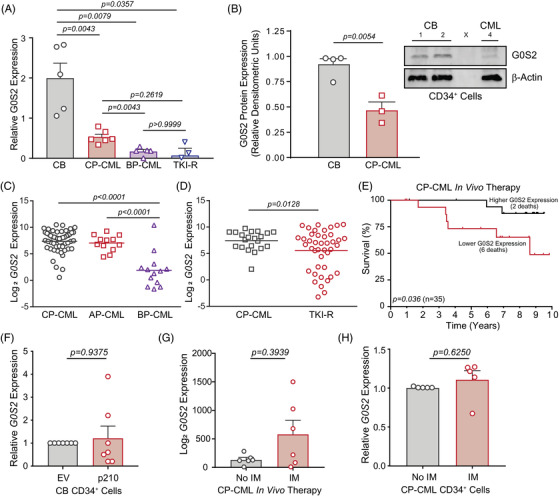
G0/G1 switch gene 2 (G0S2) is downregulated in chronic myeloid leukaemia (CML) disease progression and imatinib resistance in a BCR::ABL1 kinase‐independent manner. (A) Bar graph shows *G0S2* mRNA levels as quantified by reverse transcription‐quantitative polymerase chain reaction (RT‐qPCR) on primary CD34^+^ cells from human cord blood (CB) (*n* = 5), chronic phase CML (CP‐CML) (*n* = 6), blast phase CML (BP‐CML) (*n* = 5) and tyrosine kinase inhibitor‐resistant (TKI‐R) patients harbouring native BCR::ABL1 (*n* = 3). (B) G0S2 protein levels were analysed by immunoblot on primary CD34^+^ cells from human CB (*n* = 4) versus CP‐CML patients (*n* = 3). β‐Actin was analysed as a loading control. Bar graph represents relative densitometric units for primary cell immunoblot data. (C and D) Dot plots from RNAseq data demonstrate reduced *G0S2* mRNA levels in mononuclear cells from BP‐CML (*n* = 13) compared with CP‐CML (*n* = 53) and accelerated phase CML (AP‐CML) (*n* = 12) patients (C), and in TKI‐resistant (*n* = 42) compared with newly diagnosed (*n* = 21) CP‐CML patients (D). (E) Kaplan–Meier curve shows relative overall survival (OS) of newly diagnosed CP‐CML patients (*n* = 35) with *G0S2* mRNA expression in CD34^+^ cells (prior to imatinib therapy) above (high, *n* = 18) or below (low, *n* = 17) the value at a bimodal separation. (F) Bar graph shows relative *G0S2* mRNA levels in CB CD34^+^ cells engineered for p210 BCR::ABL1 ectopic expression (*n* = 7) versus the empty vector (EV) control (*n* = 7). (G) Bar graph demonstrates published microarray data showing *G0S2* mRNA expression levels in CP‐CML CD34^+^ cells before and after in vivo imatinib (IM) therapy (400 mg daily) for 7 days (*n* = 6) (https://www.ncbi.nlm.nih.gov/geoprofiles, GDS3518, 213524_s_at, *p* = .3939).^39^ (H) Bar graph shows *G0S2* mRNA expression in primary CP‐CML CD34^+^ cells (*n* = 5) cultured ex vivo ± IM (1 µM, 24 h) as assessed by RT‐qPCR. *GUS* mRNA levels were used as a loading control. Error bars represent standard error of the mean (SEM).

A gene expression classifier was reported to predict a patient's imatinib response after 12 months of therapy.^12^ *G0S2* mRNA was substantially downregulated in both imatinib non‐responders who lack kinase domain mutations (GSE14671, Figure S1A),^12^, ^35^ and in BP‐CML versus CP‐CML patients in another study (E‐MEXP‐480, Figure S1B).^36^ An independent dataset comparing CD34^+^ cells from normal versus CP‐CML patient BM showed consistent results (GDS2342, Figure S1C).^37^ In contrast, there was no difference in *G0S2* expression comparing CD34^+^ BM cells from healthy volunteers with that of CP‐CML patients who reached major molecular remission during imatinib therapy (GDS838, Figure S1D).^38^ We further confirmed *G0S2* downregulation in CML by RT‐qPCR analyses. These data demonstrated a 3.8‐fold reduction of *G0S2* expression in CD34^+^ cells from newly diagnosed CP‐CML patients compared with normal CB, with further downregulation by 3.1‐fold in myeloid BP‐CML (Figure 1A). While CML patients with kinase‐independent TKI resistance had reduced *G0S2* expression compared with normal CB, there was no significant difference compared with CP‐CML or BP‐CML patients (Figure 1A). Immunoblotting confirmed the downregulation of G0S2 protein in CD34^+^ cells from CML patients compared with normal CB (Figure 1B). RNAseq data on an independent patient cohort showed reduced *G0S2* expression in patients with myeloid BP‐CML and kinase‐independent TKI resistance (Figure 1C,D). Finally, lower *G0S2* expression in CD34^+^ cells from a TKI‐naïve CP‐CML cohort was associated with worse overall survival (Figure 1E). Collectively, G0S2 is downregulated in CML disease progression and TKI resistance, which correlates with worse outcomes.

We hypothesised that loss of G0S2 in CML was a direct effect of BCR::ABL1 kinase activity. Surprisingly, forced expression of p210^BCR::ABL1^ in normal CB CD34^+^ cells resulted in no significant change in *G0S2* mRNA expression (Figure 1F). *G0S2* mRNA expression was unchanged in CML patients following 7 days of in vivo imatinib therapy (GDS3518, Figure 1G)^39^ or when CML CD34^+^ cells were cultured ex vivo in the presence of imatinib (Figure 1H). Altogether, these data suggest that *G0S2* downregulation in CML and TKI resistance is independent of BCR::ABL1 kinase activity.

### 3.2 MYC/MAX mediates transcriptional repression of *G0S2* in CML

**FIGURE 2:**
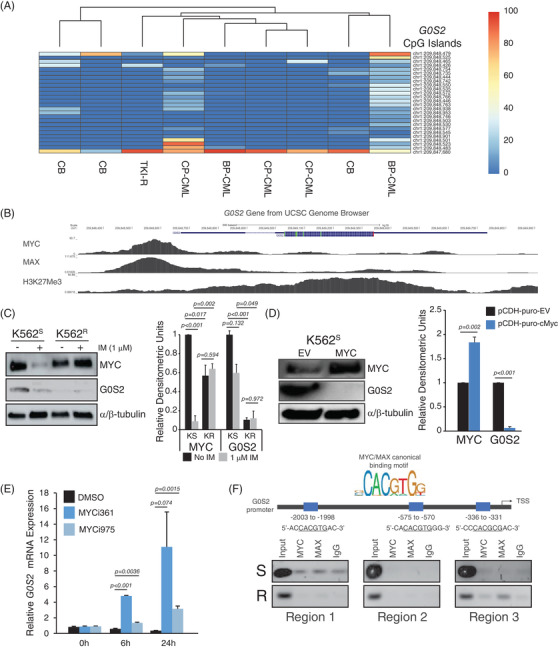
MYC/MAX mediates transcriptional repression of G0/G1 switch gene 2 (*G0S2*) in tyrosine kinase inhibitor‐resistant (TKI‐R) chronic myeloid leukaemia (CML). (A) The heat map represents CpG dinucleotide methylation in the *G0S2* promoter region as detected by DNA bisulphite conversion and patch polymerase chain reaction (PCR) sequencing on DNA of CD34^+^ cells from cord blood (CB) (*n* = 3) and CML patients (*n* = 6). (B) Tracks from the University of California at Santa Cruz Genome Browser (https://genome.ucsc.edu/) show a high degree of binding by MYC and MAX at the human *G0S2* promoter in K562 cells. (C) Immunoblot shows elevated MYC and decreased G0S2 protein expression in TKI‐R K562^R^ cells compared with TKI‐sensitive K562^S^ controls in the presence of imatinib (IM, 1 µM, 24 h). α/β‐Tubulin was assessed as a loading control. Bar graph represents relative densitometric units for *n* = 3 replicates of the experiment. (D) Immunoblot shows the level of MYC and G0S2 protein in K562^S^ cells engineered for MYC overexpression. α/β‐Tubulin was assessed as a loading control. Bar graph represents the relative densitometric units for *n* = 3 replicates of the experiment. (E) Bar graph shows relative *G0S2* mRNA expression in K562 cells treated with the MYC inhibitors, MYCi361 or MYCi975 (6 µM) for 0, 6 and 24 h. (F) MYC consensus binding sites were mapped onto the *G0S2* promoter, and the presence of MYC or MAX at the relevant site was detected by chromatin immunoprecipitation (ChIP)‐PCR (*n* = 3). Error bars represent standard error of the mean (SEM). BP‐CML, blast phase CML; CP‐CML, chronic phase CML; EV, empty vector

The *G0S2* promoter is methylated and silenced in multiple different cancers,^40^, ^41^, ^42^ including the K562 CML cell line.^43^ Consistently, treatment of BP‐CML cell lines and primary cells with the DNA methyltransferase inhibitor, 5‐azacytidine, increased *G0S2* mRNA expression (Figure S2). However, DNA bisulphite conversion and patch PCR sequencing^30^ revealed no CpG dinucleotide methylation near the *G0S2* promoter in primary CD34^+^ cells (Figure 2A), suggesting alternative mechanisms for *G0S2* downregulation in CML. ENCODE ChIP‐sequencing datasets (University of California, Santa Cruz Genome Browser) demonstrated that MYC/MAX occupies a region upstream of the *G0S2* transcription start site (TSS, Figure 2B). MYC is a nuclear phosphoprotein that regulates a variety of cellular functions, including cell growth, cell cycle, apoptosis and lipid metabolism.^44^, ^45^ MYC also plays a role in CML disease progression, LSC survival and TKI resistance.^46^, ^47^ We previously generated TKI‐resistant K562 cells (K562^R^) that are adapted for growth in the continuous presence of 1 µM imatinib and harbour native BCR::ABL1.^48^ MYC expression was upregulated in TKI‐resistant K562^R^ versus parental K562^S^ cells in the presence of imatinib, which correlated with reduced G0S2 protein expression (Figure 2C). These data suggest that MYC expression is BCR::ABL1‐dependent in TKI‐sensitive cells, but BCR::ABL1‐independent in TKI‐resistant cells. We hypothesised that MYC binds the *G0S2* promoter to repress its transcription, as previously reported for other genes.^49^ Consistently, ectopic expression of MYC in parental K562 cells rapidly reduced G0S2 protein expression (Figure 2D), whereas MYC inhibition led to rapid upregulation of *G0S2* mRNA in K562 cells (Figure 2E). To determine whether this effect was direct or indirect, we mapped MYC binding sites within the *G0S2* promoter, and performed ChIP using anti‐MYC and anti‐MAX antibodies compared with an IgG control in K562^R^ versus K562^S^ cells. MYC/MAX was enriched in region 3 upstream of the *G0S2* TSS (Figure 2F). These data indicate that upregulation of MYC is at least in part responsible for reduced G0S2 expression in CML disease progression and TKI resistance.

### 3.3 G0S2 expression impairs survival without affecting apoptosis in CML

**FIGURE 3:**
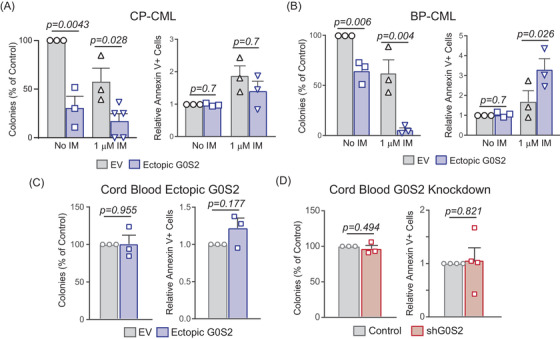
G0/G1 switch gene 2 (G0S2) acts as a tumour suppressor in primary chronic myeloid leukaemia (CML) CD34^+^ cells by impairing survival without affecting apoptosis. (A and B) Primary CD34^+^ cells isolated from chronic phase CML (CP‐CML) (A, *n* = 5) or blast phase CML (BP‐CML)/tyrosine kinase inhibitor (TKI)‐resistant (B, *n* = 3) patients were lentivirally transduced for ectopic G0S2 expression followed by colony formation (left) and apoptosis (right) assays. (C and D) Primary CD34^+^ cells isolated from normal cord blood (CB) (*n* = 3) were lentivirally transduced for G0S2 ectopic expression (C) or knockdown (D) followed by colony formation (left) and apoptosis (right) assays. Error bars represent standard error of the mean (SEM). EV, empty vector; IM, imatinib

To determine whether G0S2 is mechanistically involved in TKI resistance or strictly a biomarker, we lentivirally transduced CML cell lines and patient samples for G0S2 ectopic expression using two separate vectors, and confirmed G0S2 upregulation by immunoblot analyses and/or RT‐qPCR (Figure S3A,B). Ectopic G0S2 expression significantly reduced colony formation of both parental K562^S^ and TKI‐resistant K562^R^ cells ± imatinib (Figure S3C). Importantly, the effect of ectopic G0S2 was significantly greater in K562^R^ versus K562^S^ cells (*p* = .022). Conversely, we obtained three separate shRNA vectors targeting G0S2 (shG0S2), and confirmed knockdown at the mRNA and protein levels (Figure S3D,E). Knockdown of G0S2 significantly increased clonogenic capacity of K562^S^ cells in both the absence and presence of graded doses of imatinib (Figure S3F). In primary cells, ectopic G0S2 expression impaired colony formation in CP‐CML and BP‐CML CD34^+^ cells cultured ex vivo ± imatinib (Figure 3A,B, left). Again, the effects of ectopic G0S2 were significantly greater in BP‐CML compared with CP‐CML (*p* = .006). Although G0S2 was reported to promote apoptosis by binding to and antagonising BCL2,^13^ ectopic G0S2 had no effect on apoptosis in newly diagnosed CP‐CML CD34^+^ cells (Figure 3A, right). However, ectopic G0S2 restored imatinib sensitivity in cells from myeloid BP‐CML patients (Figure 3B, right). In normal CB CD34^+^ cells, neither G0S2 ectopic expression nor knockdown had any effect on colony formation or apoptosis (Figure 3C,D). Altogether, these data implicate a tumour suppressor role for G0S2 in CML disease progression and TKI resistance.

### 3.4 Altered G0S2 expression impairs growth of CML cells in vivo

**FIGURE 4:**
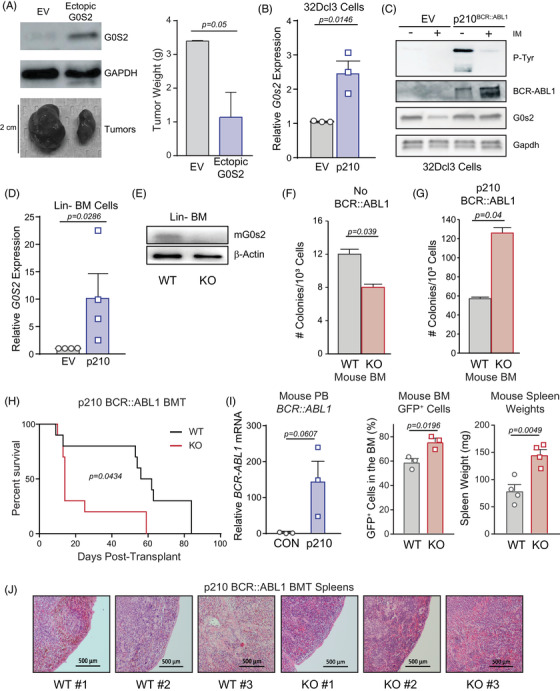
Altered G0/G1 switch gene 2 (G0S2) expression impairs growth of chronic myeloid leukaemia (CML) cells in vivo. (A) K562 cells were lentivirally transduced for ectopic G0S2 expression versus the empty vector (EV) control, and 3 × 10^6^ resulting cells were injected subcutaneously into the rear flanks of 6–8‐week‐old nude mice (*n* = 3 per group). Image shows relative size of subcutaneous tumours excised from recipient mice, and immunoblot analyses confirmed ectopic G0S2 expression at the protein level in vivo. Bar graph shows the tumour weight (g) for *n* = 3 replicates of the experiment. (B and C) Reverse transcription‐quantitative polymerase chain reaction (RT‐qPCR) (B) and immunoblot (C) analyses demonstrate murine *G0s2* mRNA and protein levels, respectively, in 32Dcl3 myeloid precursors upon ectopic expression of p210^BCR::ABL1^. (D) Similarly, RT‐qPCR shows *G0s2* mRNA in lineage‐negative mouse bone marrow (BM) upon ectopic expression of p210^BCR::ABL1^. (E) Immunoblot shows murine G0s2 protein levels in lineage‐negative mouse BM from wild‐type (WT) versus G0s2^−/−^ [knockout (KO)] mice. β‐Actin was assessed as a loading control. (F and G) Lineage‐negative mouse BM from WT or G0s2^−/−^ mice were either plated directly in colony formation assays (F) or were retrovirally transduced with p210^BCR::ABL1^ followed by plating in colony formation assays (G). Bar graphs represent the number of colonies per 1000 cells. (H) Lineage‐negative mouse BM from WT or G0s2^−/−^ mice were retrovirally transduced with p210^BCR::ABL1^ followed by intravenous injection into lethally irradiated recipients (*n* = 10 per group). Survival over time is shown in the Kaplan–Meier curve. (I) RT‐qPCR confirmed *BCR::ABL1* mRNA expression in the peripheral blood of recipient mice (left). Bar graphs show GFP^+^BCR::ABL1^+^ cells comparing recipients of WT versus G0S2^−/−^ BM cells (middle), as well as spleen weights of moribund mice after euthanasia (right). (J) Images show spleen morphology as assessed by haematoxylin and eosin staining at 4 weeks post‐transplant (*n* = 3 per group). Error bars represent standard error of the mean (SEM). BMT, bone marrow transplantation; EV, empty vector; PB, peripheral blood

In a previous report, ectopic G0S2 expression reduced subcutaneous tumour formation by K562 cells in vivo, and we confirmed these findings (Figure 4A).^43^ To assess the role of murine G0s2 in mouse models, we ectopically expressed p210^BCR::ABL1^ in murine 32Dcl3 myeloid progenitors or lineage‐negative mouse BM. In contrast with our data in human CB CD34^+^ cells (Figure 1F), enforced BCR::ABL1 expression in murine cells increased *G0s2* mRNA but not protein expression (Figure 4B,C). This is not surprising, as introduction of BCR::ABL1 into murine myeloid progenitors is known to induce cell cycle progression,^50^, ^51^ and *G0s2* was first identified as a gene that is upregulated during G0‐to‐G1 cell cycle transition in murine MNCs, hence its name.^52^, ^53^ Consistent results were observed in lineage‐negative mouse BM cells transduced with p210^BCR::ABL1^ (Figure 4D). To assess the in vivo effects of G0s2 loss in CML, we utilised lineage‐negative BM from wild‐type versus G0s2^−/−^ mice, and performed a retroviral transduction/transplantation assay that mimics BP‐CML. Ablation of G0s2 protein in the lineage‐negative BM fraction of G0s2^−/−^ mice was confirmed by immunoblot (Figure 4E). In untransformed cells, we observed a 35% reduction of colony formation in lineage‐negative G0s2^−/−^ BM cells compared with wild‐type controls (Figure 4F). Similar effects were observed in 32Dcl3 cells expressing shRNA targeting G0s2 (shG0s2) cultured in murine IL‐3, with a greater reduction of colony formation in cells cultured with mG‐CSF (Figure S4A). When we transduced lineage‐negative BM cells with p210^BCR::ABL1^, loss of G0s2 resulted in a significant increase in colony formation compared with controls (Figure 4G). Consistently, we observed a significant reduction in overall survival comparing recipients of G0s2^−/−^ versus wild‐type BM expressing the p210^BCR::ABL1^ oncoprotein (Figure 4H). Confirming that our recipient mice indeed died of leukaemia, *BCR::ABL1* transcripts were detectable in the peripheral blood of recipient mice but not controls (Figure 4I, left). Additionally, the percentage of GFP^+^ cells in the BM of moribund mice was higher in G0s2^−/−^ compared with wild‐type recipients (Figure 4I, middle), which correlated with increased splenomegaly (Figure 4I, right) and splenic cellularity (Figure 4J). Altogether, loss of G0S2 expression reduced overall survival in both CP‐CML patients (Figure 1F) and murine models of CML (Figure 4H–J).

### 3.5 *G0S2* expression correlates with myeloid development and is downregulated in the CML GMP population

**FIGURE 5:**
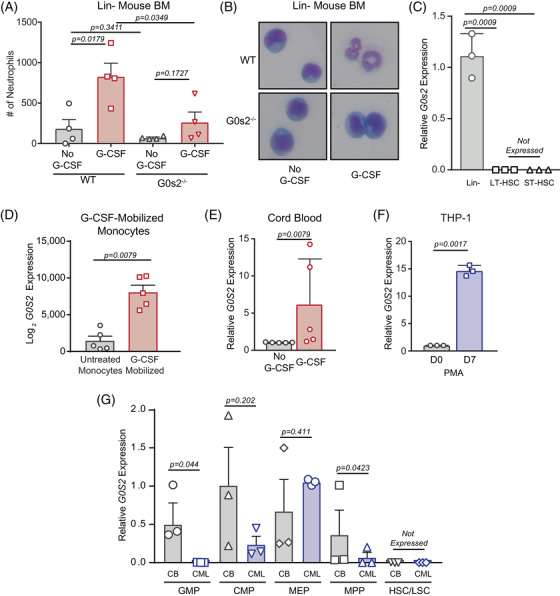
G0/G1 switch gene 2 (*G0S2*) expression correlates with myeloid development, and reduced expression in chronic myeloid leukaemia (CML) occurs within the granulocyte–macrophage progenitor (GMP) population. (A and B) Bar graph shows the number (#) of neutrophils counted in Wright–Geimsa stains of lineage‐negative (Lin^−^) mouse bone marrow (BM) from wild‐type or G0s2^−/−^ mice induced towards neutrophil differentiation with murine granulocyte‐colony stimulating factor (mG‐CSF) (A). The image shows representative morphology for the indicated treatment conditions (B). (C) Bar graph represents relative *G0s2* mRNA expression in the bulk Lin^−^ BM fraction compared with sorted long‐term (LT) and short‐term (ST) haematopoietic stem cells (HSCs) (*n* = 3). (D) Bar graph shows *G0S2* mRNA expression in haematopoietic cells after in vivo mobilisation with G‐CSF for 7 days (GSE1746, *n* = 5). (E and F) Bar graphs show *G0S2* mRNA expression in cord blood (CB) CD34^+^ cells induced to differentiate with human G‐CSF (hG‐CSF) (E, *n* = 5), or THP‐1 cells induced to differentiate with phorbol 12‐myristate 13‐acetate (PMA) for 7 days (F, *n* = 3). (G) Bar graph shows relative *G0S2* mRNA expression in CD34^+^ cells from normal CB (*n* = 3, left) or chronic phase CML (CP‐CML) patients (*n* = 3, right) that were sorted for GMPs, common myeloid progenitors (CMPs), megakaryocyte–erythrocyte progenitors (MEPs), multipotent progenitors (MPPs) or HSCs based on cell surface molecule expression. *G0S2* mRNA expression was universally low in normal HSCs and CP‐CML leukaemic stem cells (LSCs), and significantly reduced in CP‐CML versus CB GMPs. Error bars represent standard error of the mean (SEM).

The reduction of colonies in 32Dcl3‐shG0s2 cells cultured in mG‐CSF (Figure S4A, right) suggested a role for G0s2 in myeloid differentiation. Consistently, differentiation of 32Dcl3 cells in the presence of mG‐CSF markedly upregulated *G0S2* expression, but not in cells expressing p210^BCR::ABL1^ (Figure S4B). 32Dcl3‐shG0s2 cells cultured in mG‐CSF and doxycycline demonstrated a block of differentiation upon morphologic examination (Figure S4C). Similar results were observed in lineage‐negative BM from G0s2^−/−^ compared with wild‐type mice cultured in mG‐CSF (Figure 5A,B). These data suggest that G0S2 expression is associated with myeloid development. Consistently, pathway enrichment analysis of the genes co‐expressed with *G0S2* in CML^12^ revealed neutrophil degranulation as the top dysregulated pathway (>12 FC, Figure S5A,B). We performed complete blood counts (CBCs) on peripheral blood of wild‐type versus G0s2^−/−^ mice, and observed a significant reduction in only the percentage of neutrophils, with a concomitant increase in lymphocytes (Figure S6A,B). Reduced peripheral blood neutrophils in G0s2^−/−^ mice were confirmed upon visual inspection of blood smears stained with Wright–Giemsa for morphology (Figure S6C). Yamada et al. reported that *G0s2* expression in mice was highest in the most primitive HSC population, and lowest in terminally differentiated cells.^14^ However, using Gene Expression Commons, a compilation of thousands of microarray datasets, murine *G0s2* mRNA expression was lowest in primitive populations, and highest in mature granulocytes, lymphocytes and natural killer cells (Figure S6D). Data from BloodSpot confirmed these results in murine haematopoietic cells (Figure S6E).^54^, ^55^, ^56^ Consistently, RT‐qPCR experiments revealed that *G0s2* mRNA expression was undetectable in purified murine long‐term HSCs (LT‐HSCs) and short‐term HSCs (ST‐HSCs) compared with the bulk lineage‐negative fraction (Figure 5C). While *G0S2* is an ATRA target gene in APL,^23^ little is known about *G0S2* expression in human stem/progenitor cells.

Consistent with the murine data, analysis of several human datasets demonstrated low *G0S2* expression in primitive HSCs, and high *G0S2* in mature granulocytes, monocytes and CD4^+^ T cells (Figure S6F).^57^, ^58^, ^59^, ^60^, ^61^ Accordingly, *G0S2* mRNA expression was upregulated in peripheral blood CD14^+^ monocytes from human G‐CSF‐mobilised versus untreated individuals (GSE1746, Figure 5D). Differentiation of CB CD34^+^ cells with hG‐CSF, or THP‐1 cells with phorbol 12‐myristate 13‐acetate, increased *G0S2* expression (Figure 5E,F). To characterise *G0S2* expression in human haematopoietic stem/progenitor cells, we sorted CD34^+^ cells by FACS for GMPs, CMPs, MEPs, MPPs, or HSCs, and measured *G0S2* expression by RT‐qPCR. In CML versus normal CD34^+^ cells, *G0S2* mRNA expression was universally low in HSCs from normal individuals and in LSCs from CP‐CML patients. Our data revealed a loss of *G0S2* expression exclusively within the GMP population (Figure 5G). Thus, the reduction of *G0S2* expression observed in CML CD34^+^ cells (Figure 1A) is due to GMPs, the disease‐causing population in BP‐CML.^62^ Altogether, these data suggest that loss of *G0S2* expression (Figure 1A) promotes the blockade of differentiation observed in BP‐CML patients.^63^, ^64^

### 3.6 The G0S2 inhibitory effect on cell survival is independent of adipose triglyceride lipase

**FIGURE 6:**
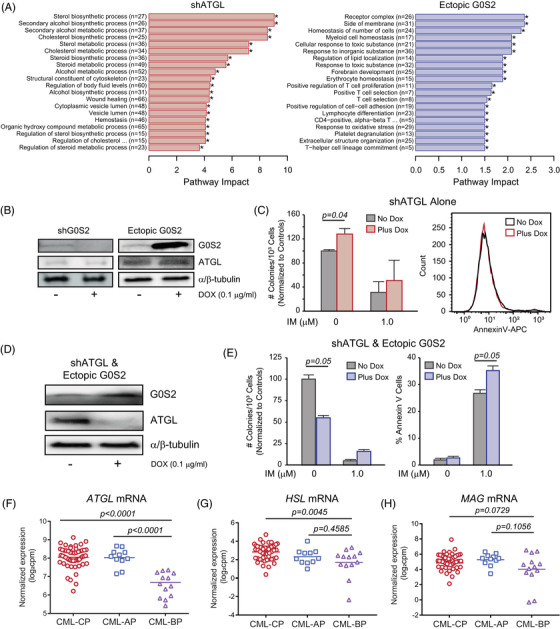
The effect of G0/G1 switch gene 2 (G0S2) on survival is independent of its known function as an inhibitor of adipose triglyceride lipase (ATGL). (A) Bar graphs show Gene Ontology (GO) analysis for the pathways dysregulated in K562^S^ cells upon ATGL knockdown (left) or ectopic G0S2 expression (right) compared with controls by RNA sequencing (*n* = 2). ^*^ *p* < .05. (B) Immunoblots show G0S2 and ATGL protein levels in K562^S^ cells upon ectopic G0S2 expression or knockdown ± doxycycline (DOX) (0.1 µg/ml, 72 h). α/β‐Tubulin was assessed as a loading control. (C) Bar graph represents colony forming ability of K562‐shATGL cells ± DOX and ± imatinib (IM) (1 µM, left). The representative histogram shows the effect of shATGL on apoptosis of K562 cells in vitro (right). (D and E) K562^S^ cells were engineered for simultaneous DOX‐inducible ATGL knockdown and ectopic G0S2 expression. Protein levels were confirmed by immunoblot analysis (D) and subject to colony formation (E, left) and apoptosis assays (E, right). (F and H) Dot plots from RNA sequencing data demonstrated reduced *ATGL* (F), hormone‐sensitive lipase (*HSL*) (G) and monoacylglycerol lipase (*MAG*) (H) mRNA levels in mononuclear cells from blast phase chronic myeloid leukaemia (BP‐CML) (*n* = 14) compared with chronic phase CML (CP‐CML) (*n* = 53) and/or accelerated phase CML (AP‐CML) (*n* = 11) patients. Error bars represent standard error of the mean (SEM).

Loss of *G0S2* expression in CML GMPs could mark a block of differentiation that promotes TKI resistance, similar to previous reports.^4^, ^65^ Gianni et al.^66^ recently published a role for lipid metabolism during ATRA‐induced differentiation of the NB4 APL cell line. It is well known that G0S2 binds to and inhibits adipose triglyceride lipase (ATGL), the rate‐limiting enzyme for intracellular lipolysis,^17^, ^20^ and G0S2‐mediated ATGL inhibition was reported to attenuate the growth of cancer cells.^67^ Lipolysis refers to the hydrolysis of triacylglycerols (TAGs) into their constituent components, including glycerol and free fatty acids (Figure S7A).^17^, ^18^ TAG fat depots are used for a number of purposes, including thermogenesis (heat), energy (fatty acid beta‐oxidation) and insulation.^68^ However, to date, the role of ATGL in CML has not been explored. Since G0S2 is an ATGL inhibitor, we hypothesised that ATGL knockdown (shATGL) would mimic ectopic G0S2 expression (Figure 3), but this was not the case. RNAseq on K562^S^ cells expressing ectopic G0S2 versus shATGL showed a clear separation between groups, but shATGL‐expressing cells did not cluster with ectopic G0S2 (Figure S8). Gene Ontology (GO) analysis of the genes dysregulated by ectopic G0S2 expression versus ATGL knockdown did not reveal concordant pathways (Figure 6A). ATGL protein is readily expressed in K562 cells, and its expression is independent of BCR::ABL1 kinase activity (Figure S7B). G0S2 ectopic expression or knockdown had no effect on ATGL protein levels in K562 cells (Figure 6B). Surprisingly, shATGL (Figure S7C) increased survival with no effect on apoptosis in K562^S^ cells and another CML cell line, KU812 (Figures 6C and S7D,E). When we ectopically expressed G0S2 upon simultaneous ATGL knockdown (Figure 6D), the phenotype in colony formation assays mimicked ectopic G0S2 alone, reducing colony formation by ∼50% (Figure 6E, left). However, ATGL knockdown combined with ectopic G0S2 expression increased imatinib‐mediated apoptosis (Figure 6E, right), similar to our observations in BP‐CML CD34^+^ cells upon ectopic G0S2 expression (Figure 3B right). Accordingly, *ATGL* mRNA was downregulated in BP‐CML versus CP‐CML or AP‐CML (Figure 6F) by RNAseq. mRNA encoding two other enzymes in the lipolytic pathway, hormone‐sensitive lipase and monoacylglycerol lipase, showed a similar trend (Figure 6G,H). These data suggest downregulation of lipolytic genes during CML disease progression, and that ATGL activity suppresses G0S2‐mediated apoptosis in the presence of imatinib.

### 3.7 Loss of G0S2 expression in CML disrupts glycerophospholipid metabolism

**FIGURE 7:**
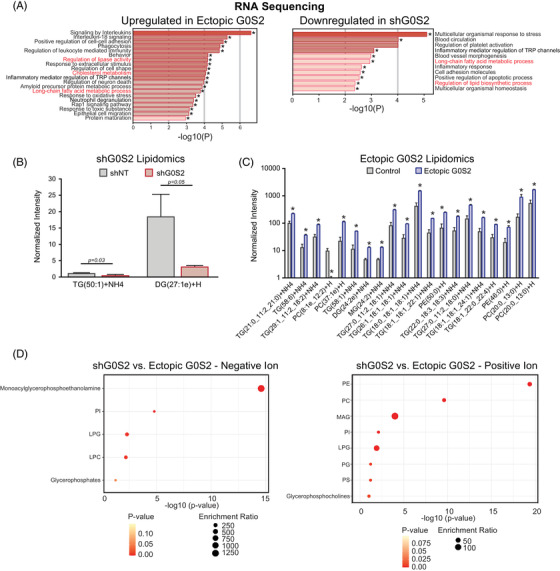
Loss of G0/G1 switch gene 2 (G0S2) expression in chronic myeloid leukaemia (CML) promotes the accumulation of very long‐chain unsaturated fatty acids and altered glycerophospholipid metabolism. (A) Bar graphs show Gene Ontology (GO) analysis for the pathways that are upregulated upon G0S2 ectopic expression (left) or downregulated upon G0S2 knockdown (right) in K562^S^ cells by RNA sequencing. ^*^ *p* < .05. Consistent pathways are indicated in red. (B and C) K562 cells expressing either the non‐targeting control vector (shNT), shRNA targeting G0S2 (shG0S2), or ectopic G0S2 were analysed by untargeted liquid chromatography (LC)/mass spectrometry (MS)‐based lipidomics. shG0S2 resulted in a reduction of very long‐chain di‐ and triglycerides (B). Ectopic G0S2 promoted the accumulation of triglycerides as well as several cell membrane components, including phosphatidylcholine (PC) and phosphatidylethanolamine (PE) species (C). (D) Lipid pathway enrichment was performed based on KEGG databases comparing K562^S^ cells with G0S2 ectopic expression or knockdown. DAG, diacylglycerol; LPC, lysophosphatidylcholine; LPG, lysophosphatidylglycerol; MAG, monoacylglycerol; PG, phosphatidylglycerol; PI, phosphatidylinositol; PS, phosphatidylserine; TAG, triacylglycerol

**FIGURE 8:**
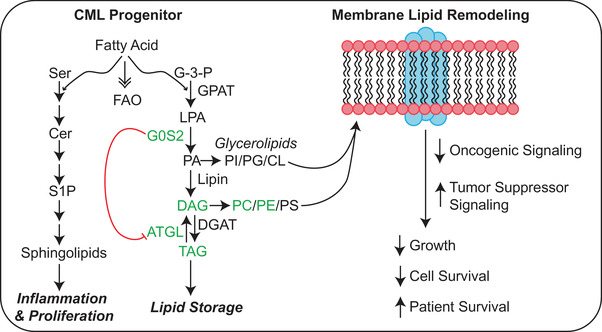
Proposed model for G0/G1 switch gene 2 (G0S2) tumour suppressor activity in chronic myeloid leukaemia (CML). The schematic shows the effect of G0S2 on triaclyglyceride (TAG) accumulation both indirectly through adipose triglyceride lipase (ATGL) inhibition, and directly through lysophosphatidic acid acyltransferase (LPAAT) activity. As ectopic G0S2 resulted in the accumulation of diacylglycerides (DAGs), TAGs, and species of phosphatidylcholine (PC) and phosphatidylethanolamine (PE) (Figure 7), our data suggest that G0S2 contributes to lipid homeostasis either by increasing TAG stores or by inducing membrane lipid remodelling. Pathway enrichment analysis of our K562 lipidomics data demonstrated that the lipid pathways affected most by differential G0S2 expression included glycerophospholipid metabolism, autophagy, glycosylphosphatidylinositol (GPI)‐anchor biosynthesis, ferroptosis and choline metabolism in cancer (Figure S9A,B). However, more work needs to be done to determine where these lipid species are being incorporated.

Thus far, our data implicate ATGL‐dependent functions for G0S2 in apoptosis of CML, and ATGL‐independent functions for G0S2 in survival of CML. The top GO pathways regulated by ectopic G0S2 expression in K562 cells included receptor complex, side of membrane and regulation of lipid localisation, among others (Figure 6A, right). Pathway enrichment analysis of differentially expressed genes comparing G0S2 ectopic expression versus knockdown in K562 cells revealed various pathways of interest. Notably, long‐chain fatty acid metabolism and other lipid pathways were upregulated in cells expressing ectopic G0S2 and downregulated in shG0S2‐expressing cells (Figure 7A). Therefore, we assessed those cells for changes in lipid species by untargeted LC/MS‐based lipidomics. Analysis of lipids altered by G0S2 revealed mono‐, di‐ and triglycerides, as expected,^17^, ^19^, ^20^ which were reduced by shG0S2 and increased by ectopic G0S2 (Figure 7B,C). We also observed increased levels of long‐chain but not short‐chain phosphatidylcholines and phosphatidylethanolamines upon ectopic G0S2 expression (Figure 7C). In fact, the most significantly different lipid categories between groups primarily included members of the glycerophospholipid family (Figures 7D and S9A,B). Glycerophospholipids consist of a polar head group attached to a glycerol backbone that includes up to two fatty acyl chains. Glycerophospholipids are characterised based on the composition of their polar head groups, including phosphatidylcholine, phosphatidylethanolamine, phosphatidylinositol, phosphatidylserine, phosphatidylglycerol and cardiolipin, and they are found mostly in membranes where they control fluidity, stability and permeability.^69^ Additional pathways regulated by G0S2 in K562 cells included autophagy, glycosylphosphatidylinositol (GPI)‐anchor biosynthesis, ferroptosis and choline metabolism in cancer (Figure S9A,B). Consistent with the reported enzymatic activity of G0S2,^19^ the phosphatidic acid PA(22:6_22:1)‐H was the top most differentially expressed lipid comparing G0S2 ectopic expression versus knockdown, with a 3.2‐fold increase in cells expressing ectopic G0S2. Thus, we focused our attention on the genes involved with these pathways in our RNAseq data. As shown in Figure S9C–H, the genes encoding acyl‐Co‐A synthetase long‐chain family member 1 (ACSL1), butyrylcholinesterase (BCHE), lysophosphatidylcholine acyltransferase 1 (LPCAT1), lysophosphatidylcholine acyltransferase 2 (LPCAT2) and phosphatidylcholine transfer protein (PCTP) were upregulated by ectopic G0S2 expression (Figure S9C–G). Conversely, the gene encoding the CD36 fatty acid transporter was downregulated by ectopic G0S2 expression (Figure S9H). Altogether, our data imply that loss of G0S2 expression in CML disrupts lipid homeostasis, particularly glycerophospholipid metabolism, resulting in a block of differentiation that renders cells resistant to TKI therapy (Figure 8).

## 4 DISCUSSION

BCR::ABL1‐positive CML is a clonal haematologic malignancy that is functionally curable through treatment with TKIs targeting BCR::ABL1.^70^ While TKI‐mediated BCR::ABL1 inhibition has revolutionised CML therapy, resistance remains a problem, and TKIs do not target the CML LSC population, requiring lifelong TKI therapy for the majority of patients.^4^ While second‐ and third‐generation TKIs were developed to combat TKI resistance, treatment‐free remission is still unattainable for many CML patients.^9^, ^70^ In the present study, we elucidated a role for G0S2 as a tumour suppressor in CML that is downregulated in multiple scenarios of TKI resistance, including TKI non‐responders versus responders (Figure 1A,D), BP‐CML versus CP‐CML (Figure 1A,C), and CP‐CML versus normal myeloid progenitors (Figure 5G). Therefore, primary TKI resistance and blastic transformation of CML may involve biologically similar pathways, as recently reported by Zhao et al.^35^ Low levels of *G0S2* mRNA correlated with worse overall survival for both CML patients (Figure 1E) and in mouse models of the disease (Figure 4H–J). G0S2 downregulation in CML was not a result of promoter hypermethylation or BCR::ABL1 kinase activity, but was rather due to transcriptional repression by MYC (Figure 2). However, we cannot rule out the possibility that loss of transcription factor expression, such as CCAAT enhancer binding protein beta (C/EBPβ)^71^, ^72^ or peroxisome proliferator‐activated receptor gamma (PPARγ),^73^, ^74^ may also play a role in reduced G0S2 expression during CML disease progression and TKI resistance. Both CEBPβ and PPARγ are known to regulate G0S2 expression in murine BM adipocytes.^16^, ^75^, ^76^, ^77^ Ultimately, our data identified a tumour suppressor role for G0S2 in CML survival and TKI resistance that was independent from its canonical function as an inhibitor of ATGL.

G0s2^−/−^ mice are born at a normal Mendelian ratio^32^; however, offspring of G0s2^−/−^ mothers do not survive 48 h due to lactation defects.^32^ Consistent with the role of G0S2 in lipolysis,^17^, ^18^ mice lacking G0s2 have defects in lactation, energy balance and thermogenesis.^32^ G0S2 function traditionally relies on protein–protein interactions, such as BCL‐2,^13^ ATGL^17^, ^67^ or nucleolin,^14^, ^43^ and G0S2‐mediated ATGL inhibition was shown to attenuate the growth of cancer cells.^67^ Although G0S2 expression alone in this study had no effect on apoptosis of CML cells, G0S2 increased imatinib‐mediated apoptosis when ATGL expression was low (BP‐CML, Figure 3B; shATGL, Figure 6E), implying that ATGL abrogates the effects of G0S2 on apoptosis. ATGL‐mediated lipolysis was shown to activate the NAD^+^‐dependent deacetylase, sirtuin 1 (SIRT1),^78^ which regulates metabolism and leukemogenic potential in CML LSCs.^79^, ^80^, ^81^, ^82^ Thus, it is possible that ATGL‐mediated SIRT1 activation could be responsible for abolishing G0S2‐mediated apoptosis in scenarios when it is highly expressed (e.g., CP‐CML). Importantly, a recent report identified an enzymatic function for G0S2 in liver hepatocytes that is independent of protein–protein interactions. These data demonstrated for the first time that G0S2 has the ability to mediate phosphatidic acid (PA) synthesis from lysophosphatidic acid and acyl‐coenzyme A, known as lysophosphatidic acid acyltransferase (LPAAT) activity.^19^ In fact, our lipidomics data on K562 cells demonstrated that PA(22:6_22:1)‐H was the top most differentially expressed lipid comparing G0S2 ectopic expression versus knockdown. Pathway enrichment analysis of our K562 lipidomics data demonstrated that the lipid pathways affected most by differential G0S2 expression included glycerophospholipid metabolism, autophagy, GPI‐anchor biosynthesis, ferroptosis and choline metabolism in cancer (Figure S9A,B). Importantly, ectopic G0S2 expression resulted in the accumulation of long‐ and very long‐chain unsaturated fatty acids in CML (Figure 7C). A recent study by Liu et al. reported that long‐chain acyl‐CoA synthetase 1 (ACSL1) overexpression enhanced the proliferation‐inhibiting effects of imatinib in CML cells.^83^ Consistently, our data suggest that *ACSL1* gene expression is upregulated by G0S2 ectopic expression in CML (Figure S8A), and therefore may play a role in its tumour suppressor activity during CML disease progression and TKI response. Future studies will explore the role of G0S2 LPAAT activity in normal and leukaemic haematopoiesis to better understand the function of G0S2 and lipid metabolism in the haematopoietic system.

G0S2 was reported to maintain quiescence of murine HSCs by sequestering nucleolin in the cytosol, thereby preventing its pro‐proliferation functions in the nucleus.^14^, ^15^ However, our data and publicly available data show that *G0S2* expression is lowest in primitive HSCs, and highest in cells committed towards the myeloid lineage (Figures 5 and S4–S6). While ectopic G0S2 binding to nucleolin could explain the reduced survival we observed in CML cells in vitro, it cannot play a role in human HSCs because G0S2 is not expressed. Rather, loss of *G0S2* expression in the GMP population predicts a block of differentiation that renders CML cells resistant to therapy. Thus, our data indicate that differentiation blockade is a unifying feature of BCR::ABL1‐independent TKI resistance, and suggest that promoting differentiation can enhance TKI responsiveness.

A major strength of our study is the use of primary CML specimens and animal models to establish G0S2 expression levels and phenotypes during CML disease progression and TKI resistance. Limitations to our study include the heavy reliance on CML cell lines for the functional and metabolic analyses. Future studies will assess the functional role of altered lipid metabolism in primary CML patient specimens and mouse models. Altogether, our data implicate G0S2 as a regulator of both myeloid differentiation and lipid metabolism pathways (Figures 5, 7 and S4–S6). RNAseq data in the current study revealed that G0S2 regulates pathways involved in fatty acid metabolism in CML, which was confirmed by LC/MS‐based lipidomics analyses (Figure 7). Thus, the role of G0S2 as a tumour suppressor in CML and in normal myeloid differentiation could depend on its LPAAT enzymatic activity and the ability to synthesise PA (Figure 8), which will be the topic of future investigation. Our data also imply that loss of G0S2 expression in CML is in part due to the MYC oncoprotein. MYC is a well‐known regulator of metabolic reprogramming in cancer,^45^ including lipid metabolism.^44^ Interestingly, lipid metabolism was recently reported to regulate ATRA‐induced differentiation of APL cells.^66^ In this study, Gianni et al. revealed that exposure of APL cells to ATRA caused an early reduction of cardiolipins, a lipid component found primarily in mitochondrial membranes.^66^ This decrease in cardiolipins was associated with inhibition of mitochondrial activity during ATRA‐induced myeloid differentiation, which they observed in ATRA‐sensitive but not ATRA‐resistant APL cells.^66^ Furthermore, lysophospholipid metabolism was recently reported to be essential for CML LSC survival.^84^, ^85^ Naka et al. demonstrated that the *Gdpd3* gene, which encodes the lysophospholipase D enzyme, is more highly expressed in murine CML stem cells compared with wild‐type HSCs, and that *Gdpd3‐*deficient CML stem cells have impaired self‐renewal capabilities.^84^, ^85^ In our study, altered G0S2 expression in CML changed the expression of di‐ and tri‐glycerides, but also several glycerophospholipids, including phosphatidylcholine and phosphatidylethanolamine (Figure 7). These changes correlated with alterations in the expression of several enzymes involved in choline metabolism, including *ACSL1*, *BCHE*, *LPCAT1/2* and *PCTP* (Figure S9). However, more work needs to be done to determine where these lipid species are being incorporated. Are they going to the cell membrane, lysosomal membranes (e.g., autophagy), endoplasmic reticulum membranes or mitochondrial membranes? Could they be contributing to enhanced mitochondrial fatty acid beta‐oxidation? These are all topics of current and future investigation in our laboratory, in order to better understand the role of lipid metabolism in CML stem cell survival and TKI response. As lipid‐modifying drugs were recently shown to enhance molecular response in CML patients on TKI therapy,^86^ our data suggest that restoring G0S2 expression and/or lipid‐modifying drugs could have clinical utility by improving lipid homeostasis, promoting myeloid differentiation and reestablishing TKI sensitivity.

## CONFLICTS OF INTEREST

B.J.D. serves on scientific advisory boards for Aileron Therapeutics, Therapy Architects (ALLCRON), Cepheid, Vivid Biosciences, Celgene, RUNX1 Research Program, Novartis, Gilead Sciences (inactive), Monojul (inactive); serves on Scientific Advisory Boards and receives stock from Aptose Biosciences, Blueprint Medicines, EnLiven Therapeutics, Iterion Therapeutics, Third Coast Therapeutics, GRAIL (inactive on scientific advisory board); is scientific founder of MolecularMD (inactive, acquired by ICON); serves on the board of directors and receives stock from Amgen, Vincera Pharma; serves on the board of directors for Burroughs Wellcome Fund, CureOne; serves on the joint steering committee for Beat AML LLS; is founder of VB Therapeutics; has a sponsored research agreement with EnLiven Therapeutics; receives clinical trial funding from Novartis, Bristol‐Myers Squibb, Pfizer. The remaining authors have no competing financial interests.

## Supporting information

Supp information
