# Supplementary material for: Loss of G0/G1 switch gene 2 (G0S2) promotes disease progression and drug resistance in chronic myeloid leukaemia (CML) by disrupting glycerophospholipid metabolism
Source: Clin Transl Med. 2022 Dec 19;12(12):e1146. doi: 10.1002/ctm2.1146 (PMC9763536; doi:10.1002/ctm2.1146)
Supplement: Supplementary file 1 — Supp information [file CTM2-12-e1146-s001.docx]

# Supporting Information

**Loss of G0/G1 switch gene 2 (G0S2) promotes disease progression and drug resistance in chronic myeloid leukemia (CML) by disrupting glycerophospholipid metabolism**

Mayra A. Gonzalez^1^ ǀ Idaly M. Olivas^1,2^ ǀ Alfonso E. Bencomo-Alvarez^1^ ǀ Andres J. Rubio^1,3^ ǀ Christian Barreto-Vargas^4^ ǀ Jose L. Lopez^3^ ǀ Sara K. Dang^2,3^ ǀ Jonathan P. Solecki^2^ ǀ Emily McCall^3^ ǀ Gonzalo Astudillo^3^ ǀ Vanessa V. Velazquez^3^ ǀ Katherine Schenkel^3^ ǀ Kelaiah Reffell^2^ ǀ Mariah Perkins^3^ ǀ Nhu Nguyen^3^ ǀ Jehu N. Apaflo^5^ ǀ Efren Alvidrez^6^ ǀ James E. Young^2^ ǀ Joshua J. Lara^2,3^ ǀ Dongqing Yan^7^ ǀ Anna Senina^7^ ǀ Jonathan Ahmann^7^ ǀ Katherine T. Varley^7^ ǀ Clinton C. Mason^7^ ǀ Christopher A. Eide^8^ ǀ Brian J. Druker^8^ ǀ Md Nurunnabi^6^ ǀ Osvaldo Padilla^9^ ǀ Sudip Bajpeyi^5^ ǀ Anna M. Eiring^1-3*^

^1^Department of Molecular and Translational Medicine, Center of Emphasis in Cancer, Texas Tech University Health Sciences Center El Paso, El Paso, TX, USA

^2^L. Frederick Francis Graduate School of Biomedical Sciences, Texas Tech University Health Sciences Center El Paso, El Paso, TX, USA

^3^Paul L. Foster School of Medicine, Texas Tech University Health Sciences Center El Paso, El Paso, TX, USA

^4^Immunology Division, University of Guadalajara, Guadalajara, Jalisco, Mexico

^5^Metabolic, Nutrition and Exercise Research (MiNER) Laboratory, Department of Kinesiology, University of Texas at El Paso (UTEP), El Paso, TX, USA

^6^Department of Pharmaceutical Sciences, School of Pharmacy, UTEP, El Paso, TX, USA

^7^Huntsman Cancer Institute, The University of Utah, Salt Lake City, UT, USA

^8^Knight Cancer Institute, Division of Hematology/Medical Oncology, Oregon Health & Science University, Portland, OR, USA

^9^Department of Pathology, TTUHSC El Paso, El Paso, TX, USA.

^*^Correspondence: Anna M. Eiring, PhD; 5001 El Paso Drive, MSC 32002, MSB1 Room 2112, El Paso, TX 79905, USA; Ph: (915) 215-4812; E-Mail: [anna.eiring@ttuhsc.edu](mailto:anna.eiring@ttuhsc.edu)

**Running Title:** G0/G1 switch gene 2 in chronic myeloid leukemia

# Supplemental Methods

***Cell lines and patient samples.*** K562, KU812, 32Dcl3, THP-1 and derivative cell lines were maintained in RPMI 1640 medium (Life Technologies, Carlsbad, CA, #21875034) supplemented with 10% fetal bovine serum (FBS, Life Technologies #16140071), 100 U/mL penicillin-streptomycin (Life Technologies #15140163), and 2 mM L-glutamine (Life Technologies #25030164). Parental K562 cells, referred to as TKI-sensitive K562^S^ cells, were purchased from American Type Culture Collection (ATCC, Manassas, VA, #CCL-243). K562^S^ cells were cultured in incrementing concentrations of imatinib (0.1- 1µM) over the course of three months to produce TKI-resistant K562^R^ cells. K562^R^ cells were cultured in the continuous presence of 1 μM imatinib (Selleck Chemicals, Houston, TX, #S1026) unless otherwise specified.^1^ To analyze *G0S2* expression upon MYC inhibition, K562^S^ were cultured at 0, 6 and 24 hours with 6 μM of MYCi361 (Selleckchem S8905), MYCi975 (Selleckchem S8906) or DMSO (Sigma, D8418). 32Dcl3 murine myeloid precursor cells were cultured in the presence of 10% WEHI-conditioned medium as a source of murine interleukin-3 (IL-3). To analyze *G0S2* expression upon monocyte differentiation, THP-1 cells were purchased from ATCC (Manassas, VA, #TIB-202) and cultured ± 200 nM phorbol 12-myristate 13-acetate (PMA, Sigma-Aldrich, St. Louis, MO, #P8139) for 7 days, as previously described,^2-4^ followed by analysis of G0S2 expression by RT-qPCR. Where indicated, 1.5x10^4^ cells were placed onto slides for Wright-Geimsa staining according to the manufacturer’s instructions (Millipore Sigma #WG16-500ML, St. Louis, MO). Patient characteristics for samples used in this study are listed in Table S1.

***Gene expression analyses using publically available data.*** Gene expression profiling was performed using the Gene Expression Omnibus (www.ncbi.nlm.nih.gov/geo/),^5,6^ Stemformatics (https://www.stemformatics.org),^7^ BloodSpot (https://servers.binf.ku.dk/bloodspot/),^8^ Oncomine (formerly available at https://oncomine.org/resource/login.html#),^9^ and Gene Expression Commons (https://gexc.riken.jp/).^10^

***Plasmids*.** For ectopic expression with a C-terminal Flag-tag (G0S2-Flag), G0S2 was PCR amplified from mononuclear cells of a healthy donor using the following primers: Fwd: 5’- CATTCTAGAGCCACCATGGAAACGGTCCAGGAGCTG-3’; Rev: 5’- ATGGCGGCCGCTTCCTACTTATCGTCGTCATCCTTGTAATCGGAGGCGTGCTGCCGGTT-3’.

G0S2-Flag was PCR purified (Qiagen) followed by subcloning into the XbaI- and NotI-digested pCDH- CMV-MCS-EF1-copGFP vector (System Biosciences, Mountain View, CA) or the doxycycline-inducible pLVX-puro vector (Clontech Laboratories, Inc., Mountain View, CA). MSCV-BCR::ABL1-IRES-GFP and the empty vector were gifts from Michael Deininger, MD, PhD, formerly at the University of Utah Huntsman Cancer Institute in Salt Lake City, UT, and currently at the Versiti Blood Research Institute (Milwaukee, WI).^11^

***Flow cytometry.*** All analyses were performed using a Guava easyCyte HT Flow Cytometer (Millipore) or a BD FACSCanto (BD Biosciences). Separation of CD34^+^38^-^ stem cells from CD34^+^38^+^ progenitor cells was achieved by FACS using a FACSAria™ II flow cytometer. To assess *G0S2* expression in stem versus progenitor cells, we sorted CD34^+^ cells from CB or CP-CML patients by FACS based on cell surface marker expression as follows: hematopoietic stem cells (HSCs), CD34^+^38^-^90^+^45RA^-^; multipotent progenitors (MPPs), CD34^+^38^+^90^-^45RA^-^; common myeloid progenitors (CMPs), CD34^+^38^+^123^+^45RA^-^; granulocyte-macrophage progenitors (GMPs), CD34^+^38^+^123^+^45RA^+^; and megakaryocyte-erythrocyte progenitors (MEPs), CD34^+^38^+^123^-^45RA^-^, as reported.^12^ Sorted cell populations were subjected to RNA extraction and analysis of *G0S2* mRNA levels by RT-qPCR. Similarly, lineage-negative murine BM cells were further sorted for long-term HSCs (CD150^+^CD48^-^) and short-term HSCs (CD150^+^CD48^+^), followed by analysis of *G0s2* mRNA levels by RT-qPCR. Antibodies for flow cytometry are listed in Supplemental Table S3.

***DNA bisulfite conversion and patch PCR sequencing.*** The human promoter sequence for *G0S2* was analyzed for CpG islands using the UCSC Genome Browser (http://www.genome.ucsc.edu) between the transcription start site (TSS) and 2000 bp upstream of the TSS. These sequences were scanned for AluI

restriction fragments, and patch oligonucleotides were designed by sequentially including base pairs from the AluI restriction sites into fragment sequences until the T_m_ of the patch oligo was between 62-67°C. Fragments whose patch oligos contained repetitive elements according to the RepeatMasker track on the UCSC Genome Browser were excluded. Patch oligos were then appended with the complement universal primer sequences, and resulting patch oligonucleotides were synthesized by SigmaGenosys (http://www.sigmaaldrich.com/Brands/Sigma_Genosys.html). Oligonucleotide sequences are listed in Supplemental Table S5. DNA bisulfite conversion (Zymo Research, Irvine, CA) and patch PCR was performed by the Molecular Diagnostics Section of the Biorepository and Molecular Pathology Shared Resource at the University of Utah, Salt Lake City, UT. DNA sequences were analyzed on a HiSeq 50 Cycle Single-Read Sequencing v4 platform at the High-Throughput Genomics Shared Resource at the University of Utah. The percent of reads that were methylated at each CG position in the target loci was calculated. CG positions with significantly different methylation percentages (p<0.05) were identified using the Kolmogorov–Smirnov test. A heatmap of differentially methylated CpG positions across samples were generated using the heatmap package version 1.0.10 in the R software package.

***Chromatin immunoprecipitation (ChIP).*** For the nuclear extract, cells were initially lysed using Farham Lysis Buffer (5 mM PIPES (pH 8), 85 mM KCl, 0.5% NP-40, 1mM DTT and fresh protease inhibitor cocktail (PIC) (Roche)). Isolated nuclei were then resuspended in Lysis Buffer (1% SDS, 10 mM EDTA, 50 mM Tris (pH 7.9), 1 mM DTT and PIC) followed by sonication to obtain 300 bp-400 bp chromatin fragments using the Bioruptor® Pico sonication device (Diagenode, Denville, NJ). Sheared chromatin was precleared with Protein A agarose beads (Millipore), followed by ChIP with 2 μg of anti-MYC (Invitrogen, Carlsbad, CA, #MA1-980,), anti-MAX (Abcam, Cambridge, United Kingdom, #ab53570), or anti-IgG (Cell Signaling, #2729) overnight at 4ºC. Protein A agarose beads were washed with TE Buffer (10 mM Tris (pH 8), 1 mM EDTA) and then blocked with 1 µg/µl of BSA (Sigma-Aldrich). To pulldown the complex, blocked beads were added, and after 2 hours of binding, were washed with Low Salt Buffer (20 mM Tris (pH 7,9), 2 mM EDTA, 125 mM NaCl, 0.05% SDS, 1% Triton x-100 and PIC), High Salt Buffer (20 mM Tris (pH 7,9), 2 mM EDTA, 500 mM NaCl, 0.05% SDS, 1% Triton x-100 and PIC), LiCl Buffer (10 mM Tris (pH 7,9), 2 mM EDTA, 250 mM LiCl, 1% NP-40, 1% Sodium Deoxycholate and PIC) and TE Buffer, consecutively. RNase A (Thermo Fisher Scientific#12091021) and Proteinase K (Thermo Fisher Scientific #25530049) were then added to the beads and kept for de-crosslinking at 65ºC. Phenol/chloroform extraction followed by isopropanol precipitation was performed, and the resulting DNA pellet was dissolved in H_2_O followed by PCR using specific primers targeting three regions within the *G0S2* promoter that contain putative binding sites for MYC/MAX. Primer sequences are listed in Supplemental Table S6.

***Animal models*.** Wild-type K562 cells were lentivirally transduced for ectopic G0S2 expression versus the empty vector control and selected in puromycin (2 μg/ml, 72 h). To generate subcutaneous tumors, 3x10^6^ cells were mixed with 100 μl of Matrigel membrane matrix (Corning Inc., Corning, NY, #356234), and injected subcutaneously into the rear flanks of 6-8 week old nude mice (Jackson Laboratory, Bar Harbor, ME). Ten days after implantation, all mice were placed on doxycycline chow (Envigo, Indianapolis, IN, #TD.01306) to induce vector expression, and tumor size was measured every three days.^13^ After 21 days, mice were euthanized and tumors removed for gross examination and molecular analyses. To evaluate the importance of G0S2 expression for BCR::ABL1 survival in murine cells, lineage-negative bone marrow from 6-week-old wild-type or G0s2^-/-^ mice (n=10 per group)^14^ were transduced with MSCV-BCR::ABL1-IRES-GFP or the empty vector (EV), and sorted GFP^-^ or GFP^+^ cells were plated in colony formation assays in the presence of recombinant murine kit ligand (10ng/ml), IL-3 (2ng/ml), IL-6 (1.2ng/ml), Flt3 ligand (5ng/ml), and GM-CSF (5ng/ml). Colonies were scored after 2 weeks. *BCR::ABL1* expression was confirmed by RT-qPCR. Where indicated, peripheral blood smears were performed and stained by Wright- Geimsa according to the manufacturer’s instructions (Millipore Sigma).

***RNA sequencing (RNAseq).*** For RNAseq analyses, two biological replicates were sequenced for each condition: shRNA non-targeting control (shNT), ectopic G0S2 (pLVX-G0S2), shRNA targeting G0S2 (shG0S2), and shRNA targeting ATGL (shATGL). All replicates were cultured in doxycycline (0.1 μg/ml, 72 h) to induce expression or knockdown. Total RNA was extracted using the PureLink RNA Mini Kit by Invitrogen (Thermo Fisher Scientific) and quantified using a NanoDrop^TM^ 2000 (Thermo Fisher Scientific). RNA was eluted with RNAse-free water and quality was assayed by electrophoresis (Agilent RNA Screentape, Agilent Technologies, Santa Clara, CA). Samples with an RNA integrity number (RIN) value above 9 were included in the analysis. RNAseq libraries were prepared and sequenced at Novogene Corporation (Beijing, China). HTSeq v0.6.1 was used to count the read numbers mapped for each gene. Differential expression analysis between groups was performed using the DESeq2 R package (2_1.6.3). Resulting p values were adjusted using the Benjamini and Hochberg’s approach for controlling the False Discovery Rate (FDR). Genes with an adjusted P value <0.05 were considered differentially expressed. Gene Ontology (GO) and Kyoto Encyclopedia of Genes and Genomes (KEGG) enrichment analysis of differentially expressed genes was implemented using the clusterProfiler R package, and GO or KEGG terms with a corrected P value of <0.05 were considered significantly enriched. Differentially expressed genes were visualized using a heatmap with default parameters using the R package.

# Supplemental Tables

## Table S1. Patient characteristics table.


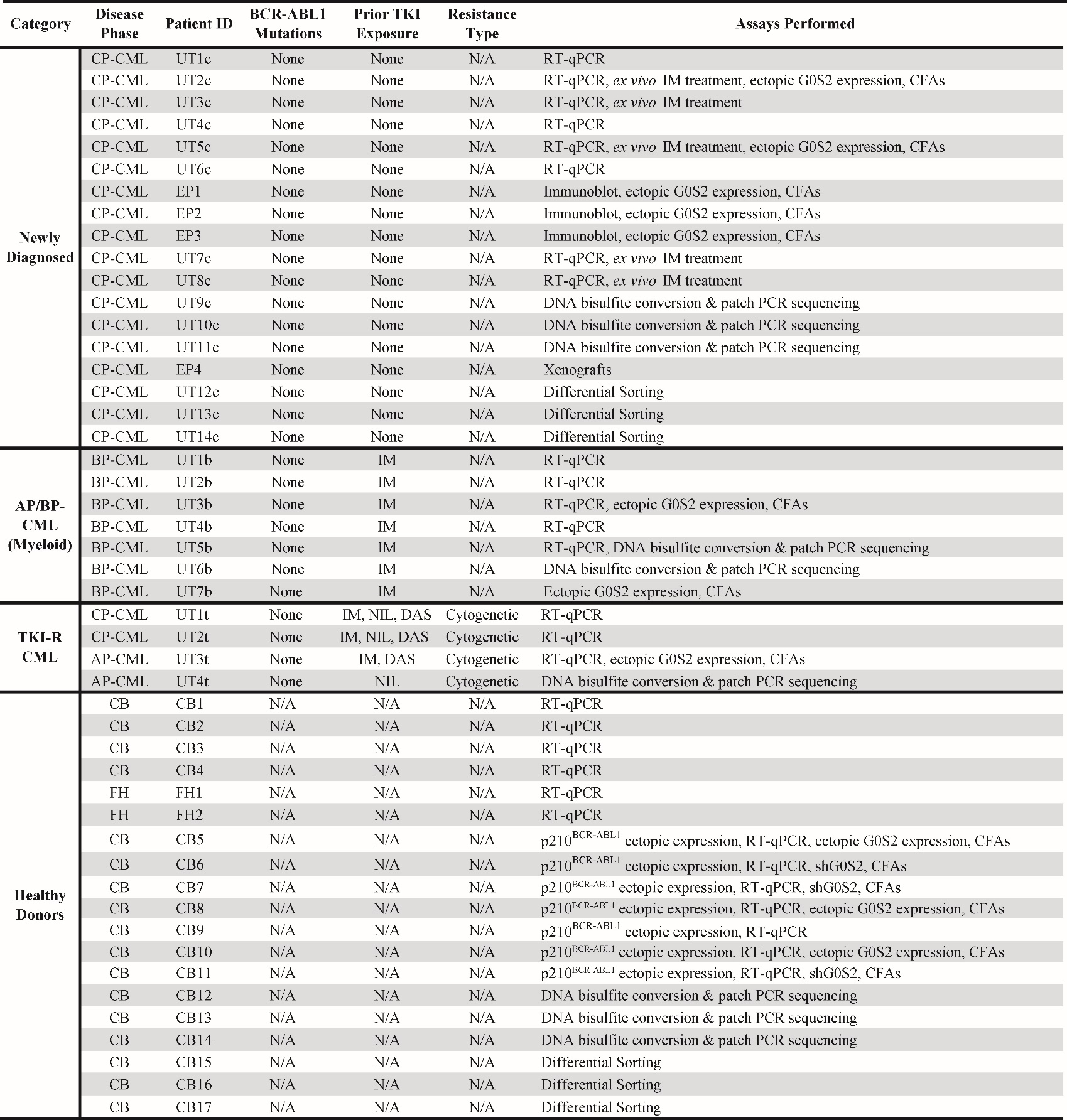


AP-CML, accelerated phase-chronic myeloid leukemia; BP-CML, blast phase-chronic myeloid leukemia; CB, cord blood; CML, chronic myeloid leukemia; CP-CML, chronic phase CML; DNA, deoxyribonucleic acid; FH, femoral head CD34^+^ cells; G0S2, G0/G1 switch gene 2; N/A, not applicable; PCR, polymerase chain reaction; RT-qPCR; reverse transcription- quantitative PCR; TKI-R, TKI-resistant CML.

## Table S2. Primers used for quantitative real-time polymerase chain reaction (RT-qPCR).

| ***Gene*** | ***Forward (5’-3’)*** | ***Reverse (5’-3’)*** |
| --- | --- | --- |
| *hG0S2* | CGTGCCACTAAGGTCATTCC | GCACGTACAGCTTCACCATC |
| *hATGL* | AGAGATGTGCAAGCAGGG | TCCTCGGCGTCTACTACGTC |
| *hGUSB* | GAAAATATGTGGTTGGAGAGCTCATT | CCGAGTGAAGATCCCCTTTTTA |
| *hBCR::ABL1* | TCCGCTGACCATCAACAAGGA | CACTCAGACCCTGAGGCTCAA |
| *mG0s2* | GGCATTGCTCTCAATGACAA | TGTGAGGGAGATGCTCAGTG |
| *mGapdh* | CTGCGGGAAGCGTGTGAAC | GTGCAGCACGCACTGTGAT |

*ATGL*, adipocyte triglyceride lipase; *BCR::ABL1*, breakpoint cluster region-abelson tyrosine-protein kinase fusion gene; *G0S2*, G0/G1 switch gene 2; *GAPDH*, glyceraldehyde-3-phosphate dehydrogenase; *GUSB*, glucuronidase-beta. (h) refers to human genes; (m) refers to murine genes.

## Table S3. Antibodies used for immunoblot or flow cytometry.

| ***Name*** | ***Vendor*** | ***Catalog #*** |
| --- | --- | --- |
| Rabbit anti-G0S2 | United States Biological | 127066 |
| Rabbit anti-α/β-Tubulin | Cell Signaling Technology | 2148 |
| Mouse anti-FLAG M2 | Sigma-Aldrich | F3165 |
| Anti-Beta-Actin | Sigma-Aldrich | A5441 |
| Anti-Phospho-Tyrosine | Cell Signaling Technology | 9411 |
| Anti-GAPDH | Cell Signaling Technology | 2118 |
| FITC-anti-CD34 | eBioscience | 11-0341-81 |
| APC-anti-CD38 | BioLegend | 102712 |
| APC-anti-hCD11b | ThermoFisher Scientific | 17-0118-41 |
| PE-Cy7-anti-hCD34 | BD Biosciences | 348791 |
| FITC-anti-hCD45RA | BD Biosciences | 347723 |
| APC-anti-hCD38 | BD Biosciences | 340439 |
| PE-anti-hCD123 | BD Biosciences | 340545 |
| BV421-anti-hCD90 | BD Biosciences | 562556 |
| PerCP/Cyanine 5.5 anti-mCD150 | BioLegend | 115922 |
| PE/Cyanine7 anti-mouse CD48 | BioLegend | 103424 |

APC, allophycocyanin; BV, brilliant violet; CD, cluster of differentiation; c-MYC, cellular myelocytomatosis gene; FITC, fluorescein isothiocyanate; GAPDH, glyceraldehyde-3-phosphate dehydrogenase; G0S2, G0/G1 switch gene 2; IgG, immunoglobulin G; PE, phycoerythrin.

## Table S4. Lentiviral vectors for virus production.

| ***Vector*** | ***Vendor*** | ***Catalog #*** |
| --- | --- | --- |
| psPAX2 | Addgene | 12260 |
| VSV.G | Addgene | 14888 |
| pCL-Eco | Addgene | 12371 |
| shG0S2 | Cellecta, Inc. | CVSHC-PX |
| pLVX-Tetone-puro-G0S2 | Clontech | 631849 |
| pCDH-puro-G0S2 | System Biosciences | CD511B-1 |
| shATGL | Cellecta, Inc. | CVSHC-PX |
| shNT | Cellecta Inc. | CVSHC-PX |
| pCDH-puro-cMyc | Addgene | 46970 |
| MSCV-IRES-GFP | Gift | N/A |
| MSCV-BCR::ABL1-IRES-GFP | Gift | N/A |

ATGL, adipose triglyceride lipase; NT, non-targeting; G0S2, G0/G1 switch gene 2; GFP, green fluorescent protein; pCL-Eco, retrovirus packaging plasmid; psPAX2, empty backbone 2^nd^ generation lentiviral packaging plasmid; pLVX, lentiviral expression plasmid; pCDH, lentiviral expression plasmid; MSCV, murine stem cell leukemia virus; shRNA, small hairpin ribonucleic acid; VSV.G, vesicular stomatitis virus G glycoprotein.

## Table S5. Patch oligonucleotides used for DNA bisulfite conversion and patch PCR sequencing.

| ***hG0S2 Region*** | ***Forward (5’-3’)*** | ***Reverse (5’-3’)*** |
| --- | --- | --- |
| *chr1:2098476 25-209847712* | ttagctgtaatcacctggggctgaACTCCCCACCTTC CTCATTCTCT | AACACCAACTACCCTCCCACACTggtaaga  atccagagtgtgagtactc |
| *chr1:2098483 98-209848506* | cttcttactggtgtcagcgggtgACTCCCCACCTTC CTCATTCTCT | AACACCAACTACCCTCCCACACTcacgcga  aggtcccactc |
| *chr1:2098485 07-209848581* | cgtgggaaggccagtgtgACTCCCCACCTTCCT CATTCTCT | AACACCAACTACCCTCCCACACTgcacgct  ccgactgtgt |
| *chr1:2098485*  *82-209848716* | gtggccacgcgctgaACTCCCCACCTTCCTCA  TTCTCT | AACACCAACTACCCTCCCACACTgacacgc  ctcctgggc |
| *chr1:2098487 17-209848789* | ggggccgcttatatcttttctctgaACTCCCCACCTTC CTCATTCTCT | AACACCAACTACCCTCCCACACTcagctctc  ccagttggagact |
| *chr1:2098488 91-209848994* | gctcatagaaaggcggactaccttaACTCCCCACCT TCCTCATTCTCT | AACACCAACTACCCTCCCACACTcaggagg  agcagcaggaga |

G0S2, G0/G1 switch gene 2.

## Table S6. Primers used for ChIP-PCR of the *G0S2* promoter containing MYC/MAX binding sites.

| **G0S2 promoter** | **MYC/MAX Binding motif** | **Forward primer (5’-3’)** | **Reverse primer (5’-3’)** | **Product Length (bp)** |
| --- | --- | --- | --- | --- |
| Region 1 | CACGTG (CANONICAL) | gcttactggaccagtcaattt | GTGAGCTTTCCCAATATCTGT | 159 |
| Region 2 | CACGTG (cANONICAL) | CTGTCCTGTTACCCAGATCCT | GCGGCAACCTTCTTACTG | 204 |
| Region 3 | CACGCG (nON-CANONICAL) | GCCTGGAGTGGGACCTTC | GGAAGAAGAGGAGGGGAAAA | 168 |

## G0S2, G0/G1 switch gene 2; MAX, MYC-associated factor X; MYC, MYC proto-oncogene.

**Table S7. Abbreviations for murine blood cell types**

| ***Abbreviation*** | ***Name*** |
| --- | --- |
| *B-Cell* | B cells |
| *CD4* | CD4 T cells |
| *CD4 activated* | Activated CD4-positive T cells |
| *CD8 activated* | Activated CD8-positive T cells |
| *CD8 naïve* | Naïve CD8-positive T cells |
| *CFUE* | Colony-forming unit erythroid cells |
| *CLP* | Common lymphoid progenitor cells |
| *ETP* | Early T cell progenitor |
| *GMP* | Granulocyte monocyte progenitors |
| *Granulocytes* | Granulocytes |
| *IgM^+^ SP* | Immunoglobulin M-positive side population cells |
| *LMPP* | Lymphoid-primed multipotential progenitors |
| *LT-HSC* | Long-term hematopoietic stem cell |
| *MkE* | Megakaryocyte erythroid precursors |
| *MkP* | Megakaryocyte precursor |
| *Monocyte* | Monocytes |
| *NK Cells* | CD56^+^ Natural killer cells |
| *NKmature* | Mature natural killer cells |
| *Nucleated Erythrocytes* | Nucleated erythrocytes |
| *PreB* | Pre-B cells |
| *PreCFUE* | Pre-colony-forming unit erythroid cells |
| *ProB* | Pro-B cells |
| *ProE* | Erythroid progenitor cells |
| *St-HSC* | Short-term hematopoietic stem cells |
| *preGM* | Pre-Granulocyte Monocytes |

**Table S8. Abbreviations for human blood cell types**

| ***Abbreviation*** | ***Name*** |
| --- | --- |
| *HSC_BM* | Hematopoietic stem cells from bone marrow |
| *Early HPC BM* | Hematopoietic progenitor cells from bone marrow |
| *CMP* | Common myeloid progenitor |
| *GMP* | Granulocyte myeloid progenitor |
| *MEP* | Megakaryocyte-erythroid progenitor cells |
| *PM_BM* | Promyelocyte from bone marrow |
| *MY_BM* | Myelocyte from bone marrow |
| *PMN_BM* | Polymorphonuclear cells from bone marrow |
| *PMN_PB* | Polymorphonuclear cells from peripheral blood |
| *CD14^+^*  *monocytes* | CD14^+^ Monocytes |
| *B cells* | CD19^+^ B cells |
| *CD4^+^ T Cells* | CD4^+^ T cells |
| *CD8^+^ T Cells* | CD8^+^ T cells |
| *NK cells* | CD56^+^ Natural killer cells |
| *mDC* | CD11c^+^ Myeloid dendritic cells |
| *pDC* | CD123^+^ Plasmacytoid dendritic cells |

# Supplemental Figures

**
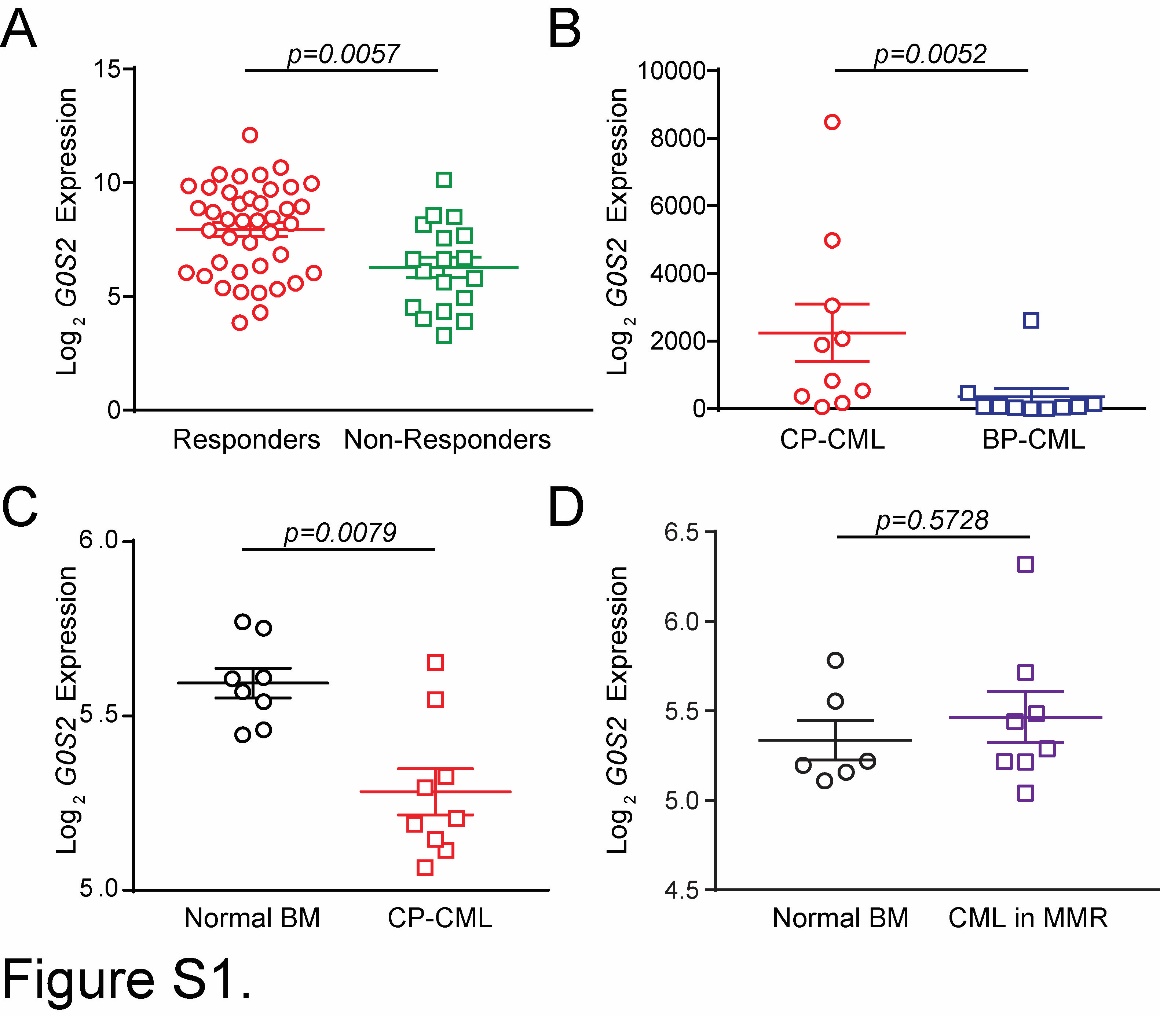
**

**Figure S1. *G0S2* mRNA expression is downregulated in TKI-resistant CML versus newly diagnosed CML, in BP-CML versus CP-CML, and in CML versus normal bone marrow (BM). A-D.** The dot plots show expression of *G0S2* mRNA (213524_s_at) in (**A**) CML patients expressing native BCR::ABL1 who either responded (responders, n=41) or did not respond (non-responders, n=18) to imatinib (GSE14671),^15^ (**B**) CD34^+^ cells from CP-CML (n=10) versus BP-CML (n=10) patients (E-MEXP-480),^16^ (**C**) CD34^+^ cells from normal (n=8) versus CML bone marrow (n=9) (GDS2342),^17^ and (**D**) bone marrow CD34^+^ cells from healthy donors (n=6) versus CML patients in major molecular remission (MMR, n=8, GDS838).^18^ Error bars represent standard error of the mean (SEM).


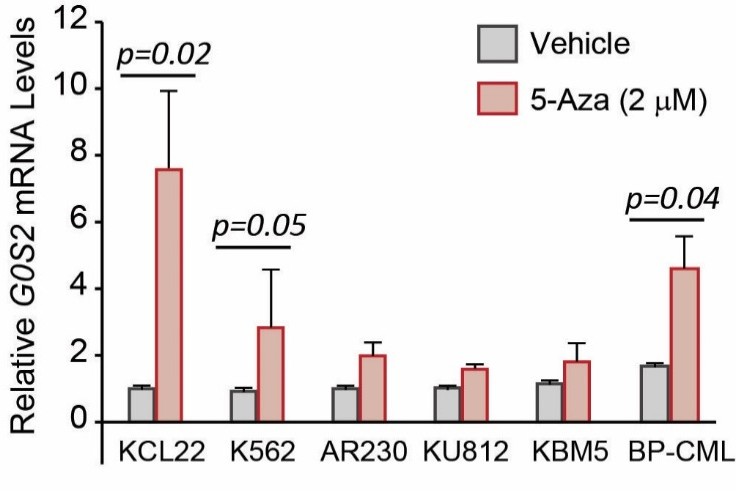


**Figure S2. Treatment of CML cells with 5-azacitidine increased *G0S2* mRNA expression. A.** Bar graph represents *G0S2* mRNA levels as measured by RT-qPCR in the indicated CML cells ± 5-azacitidine (5-Aza, 2 μM, 72h, n=3/group). *GUS* was measured as a loading control. Error bars represent SEM.


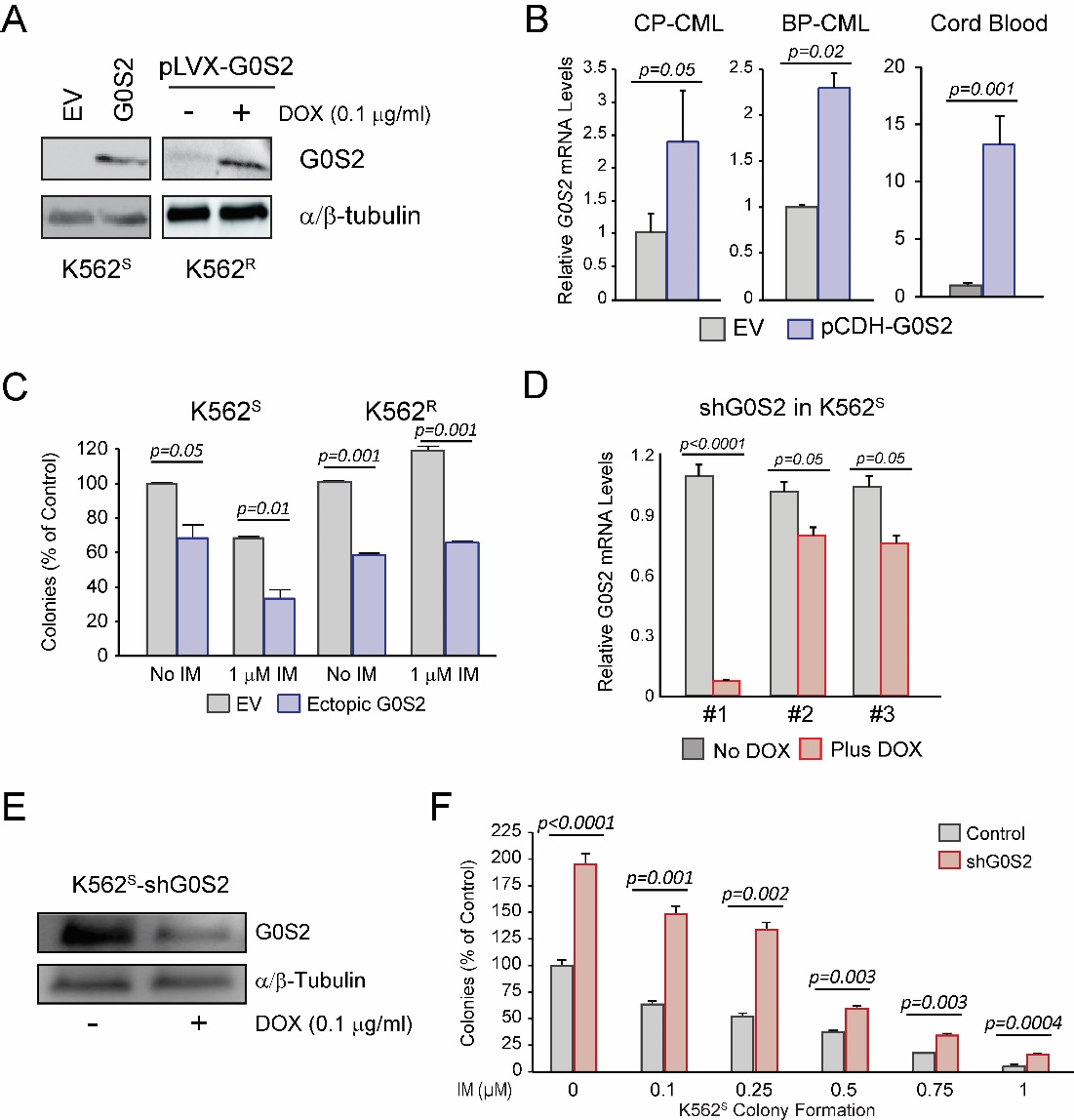


**Figure S3. G0S2 ectopic expression or knockdown in CML cell lines and primary CD34^+^ cells from CML patients and normal cord blood. A.** Ectopic G0S2 expression in TKI-sensitive K562^S^ cells and TKI-resistant K562^R^ cells was confirmed by immunoblot analyses using an anti-FLAG antibody. Two different vectors were used, including pCDH-CMV-MCS-EF1-copGFP vector (System Biosciences, *left*), which can be selected for green fluorescent protein (GFP)-positive cells, or the doxycycline-inducible pLVX-puro vector (Clontech Laboratories, *right*), which can be selected by culture in puromycin (n=3/group). **B.** Ectopic G0S2 expression in primary CD34^+^ cells from CML patients or normal cord blood was confirmed by RT-qPCR. *G0S2* mRNA levels were normalized to *GUSB* (n=3/group). **C.** Bar graph shows relative colony forming units for parental K562^S^ cells (n=3) and TKI-resistant K562^R^ cells (n=3) lentivirally transduced for ectopic G0S2 expression. **D.** K562^S^ cells were lentivirally transduced with three different shRNA constructs targeting *G0S2* (shG0S2), and resulting cells were cultured ± doxycycline (0.1 μg/ml, 72 h) to induce the knockdown. RT-qPCR was used to assess *G0S2* mRNA levels following culture with and without doxycycline. *GUS* mRNA was measured as a control (n=3/group). **E.** Immunoblot shows G0S2 protein levels in K562^S^ cells expressing shG0S2 ± doxycycline (0.1 μg/ml, 72 h) to induce the knockdown (n=3/group). **F.** Bar graph shows the effect of shG0S2 on colony formation of K562^S^ cells in graded concentrations of imatinib ± doxycycline (0.1 μg/ml) to induce the knockdown (n=3/group). Error bars represent SEM.


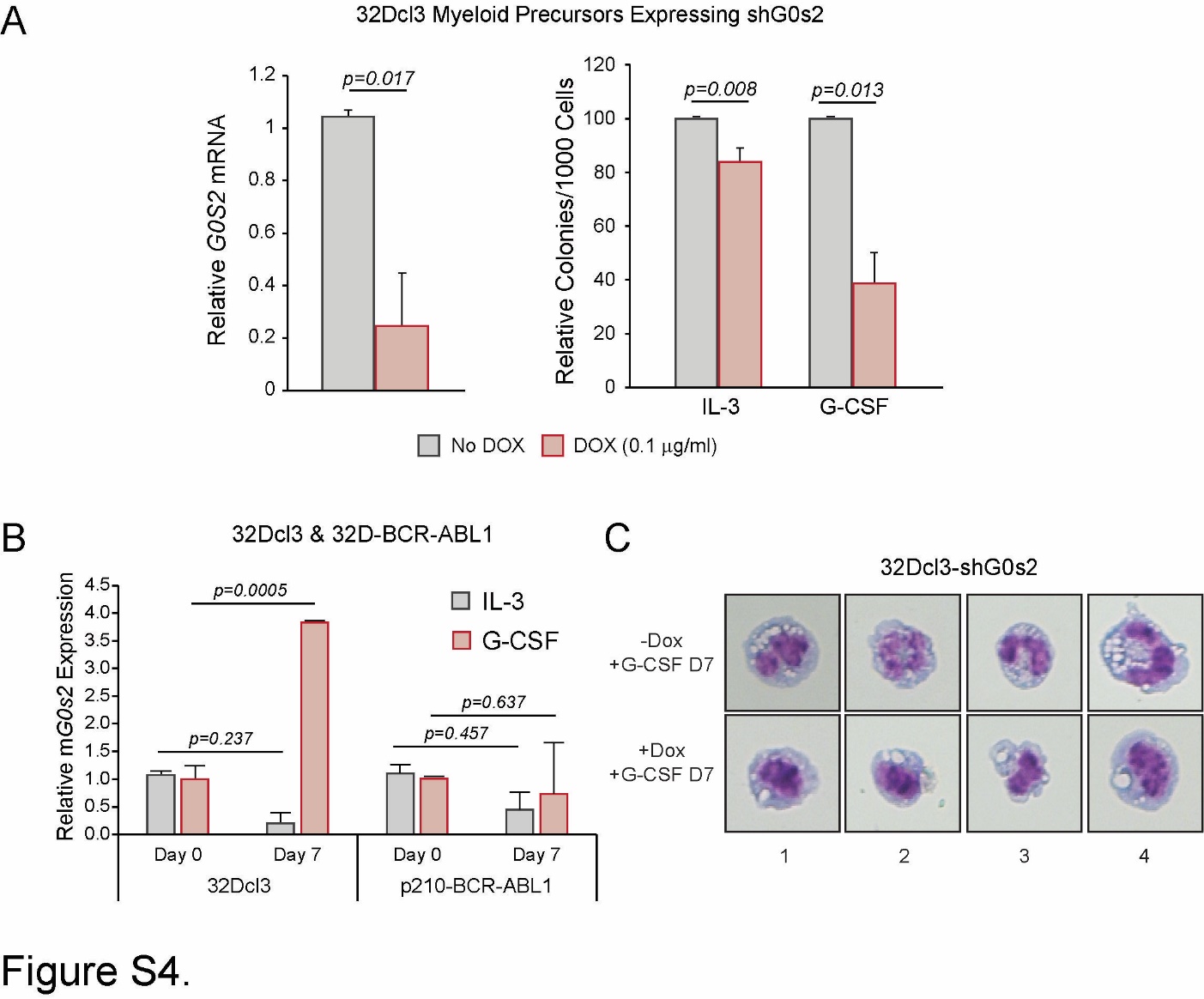


**Figure S4. *G0S2* expression associates with myeloid development. A.** 32Dcl3 murine myeloid precursor cells were lentivirally transduced with shG0s2; bar graph represents *G0s2* mRNA expression ± doxycycline (0.1 mg/ml, 72h) as measured by RT-qPCR (*left*). Resulting cells were plated in colony formation assays in the presence of murine interleukin-3 (mIL-3) or granulocyte-colony stimulating factor (mG-CSF), and scored after 7 days (bar graph on the *right*, n=3/group). **B.** Bar graph shows *G0s2* mRNA expression in the murine 32Dcl3 myeloid progenitor cell line transduced with p210 BCR::ABL1 or the empty vector, and treated with IL-3 or mG-CSF for 7 days (n=3). **C.** A block of differentiation in 32Dcl3 cells expressing shG0s2 was confirmed by Wright-Giemsa staining for morphology (n=4). IL-3, interleukin-3; G-CSF, granulocyte-colony stimulating factor; mG0s2, murine G0/G1 switch gene 2.

**
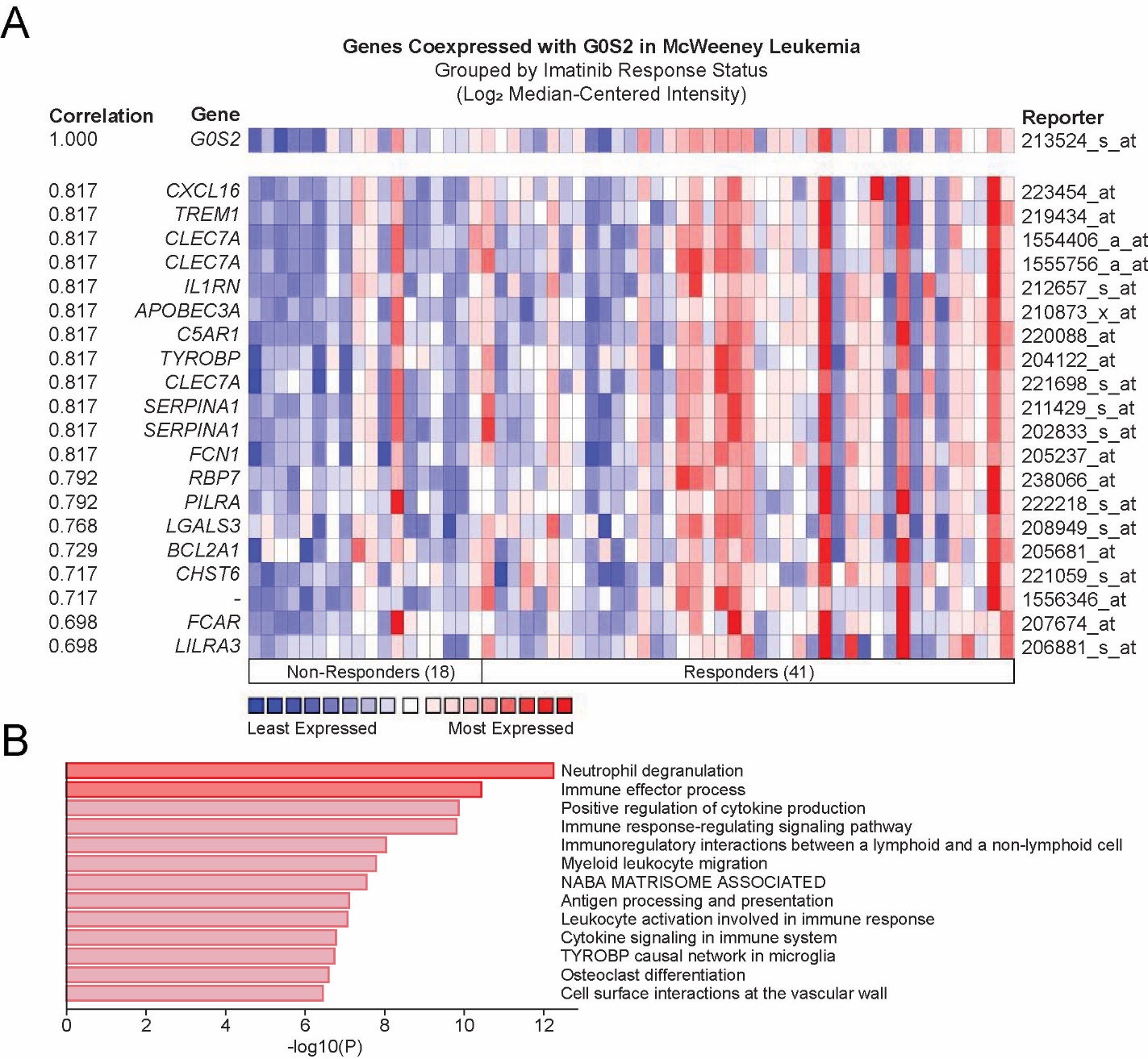
**

**Figure S5. The genes co-expressed with *G0S2* in TKI responders versus non-responders correlate with neutrophil degranulation. A.** Using data previously available from Oncomine, the heat map shows the top genes that are co-expressed with *G0S2* in CML CD34^+^ cells from TKI responders versus non-responders in McWeeney et al.^15^ **B.** The bar graph shows pathway enrichment analysis for the genes co-expressed with *G0S2* in CML TKI resistance. The top most differentially expressed pathway involved neutrophil degranulation.


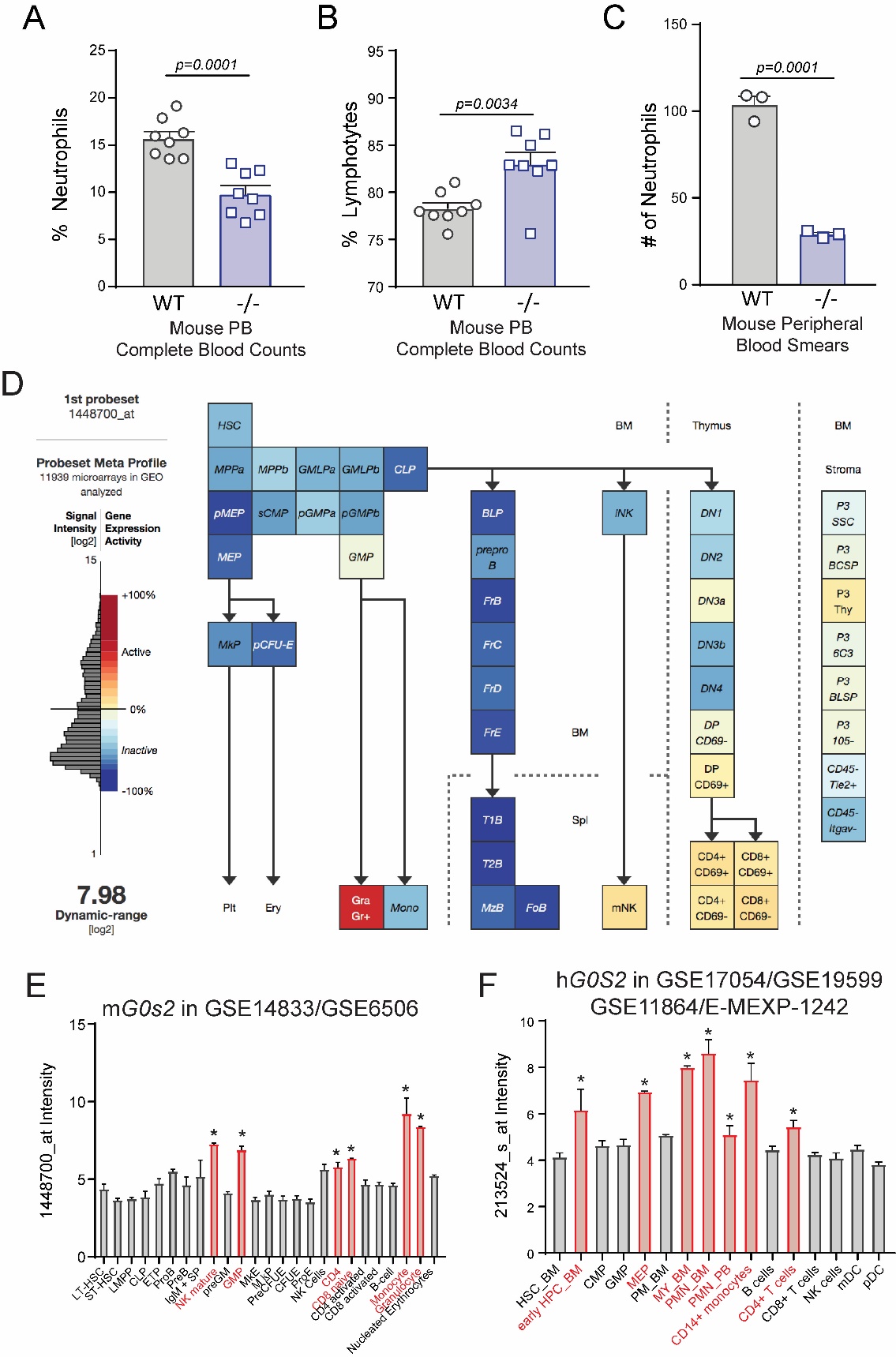


**Figure S6. *G0S2* and myeloid development in different mouse and human blood cell types. A-B.** Bar graphs show percent (%) neutrophils (**A**) and lymphocytes (**B**) in complete blood counts (CBCs) comparing peripheral blood from wild-type versus G0s2^-/-^ knockout mice (n=8/group). No significant defects in other blood parameters were noted. **C.** Bar graph shows the number (#) of neutrophils observed in peripheral blood smears from wild-type versus G0s2^-/-^ knockout mice (n=3/group). **D.** Data from Gene Expression Commons (https://gexc.riken.jp/) analyzing 11,939 microarray datasets revealed that murine *G0s2* mRNA expression is highest in granulocyte-macrophage progenitors (GMPs) and mature granulocytes, lymphocytes, and natural killer cells. **E-F.** Bar graphs show probe intensity for murine *G0s2* (1448700_at) in GSE14833 and GSE6506 (**E**)^19-21^ and human *G0S2* (213524_s_at) in GSE17054, GSE19599, GSE11864, and E-MEXP-1242 (**F**).^22-26^ Raw data were downloaded from https://BloodSpot.eu. Abbreviations for panels **E-F** are available in Supplemental Tables S7-8. Red bars indicate statistical significance at *p<0.05.


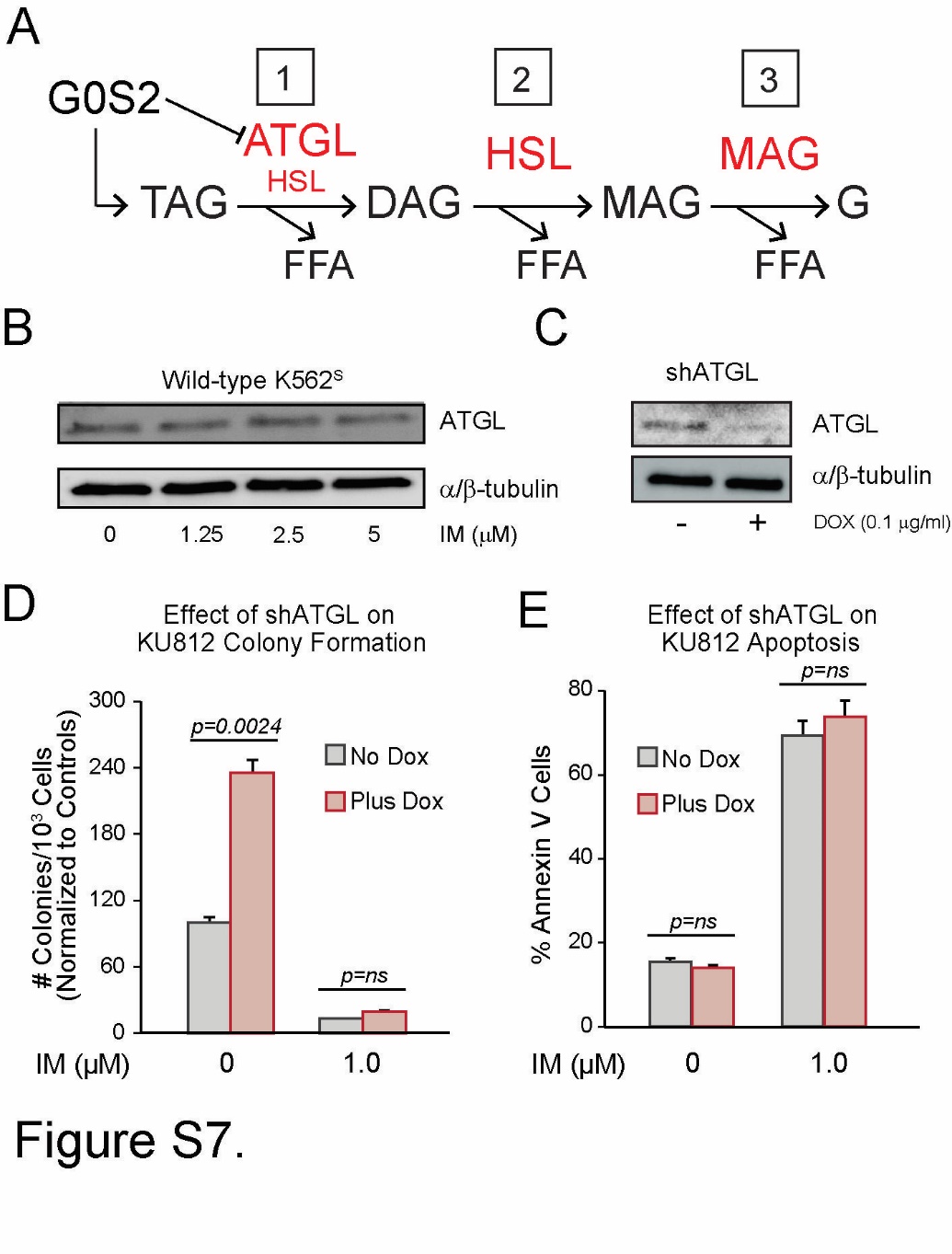


**Figure S7. The effects of G0S2 in CML are independent from its role as an inhibitor of ATGL. A.** The schematic shows the inhibitory effect of G0S2 on ATGL, and the catabolic activity of G0S2 in triglyceride (TAG) production. ATGL, adipocyte triglyceride lipase; DAG, diacylglycerol; FFA, free fatty acid; G, glycerol; G0S2, G0/G1 switch gene 2; HSL, hormone sensitive lipase; MAG, monoacylglycerol; TAG, triacylglycerol. **B-C.** Immunoblots show ATGL protein expression in K562 cells either untreated or treated with graded concentrations of imatinib (24 h, **B**), or in K562 cells expressing shRNA targeting ATGL (shATGL) ± doxycycline (0.1 μg/mL, 72 h, **C**) (n=3/group). **D-E.** Bar graphs show the effects of shATGL on colony forming ability (**D**) or apoptosis (**E**) in the KU812 CML cell line (n=3/group).


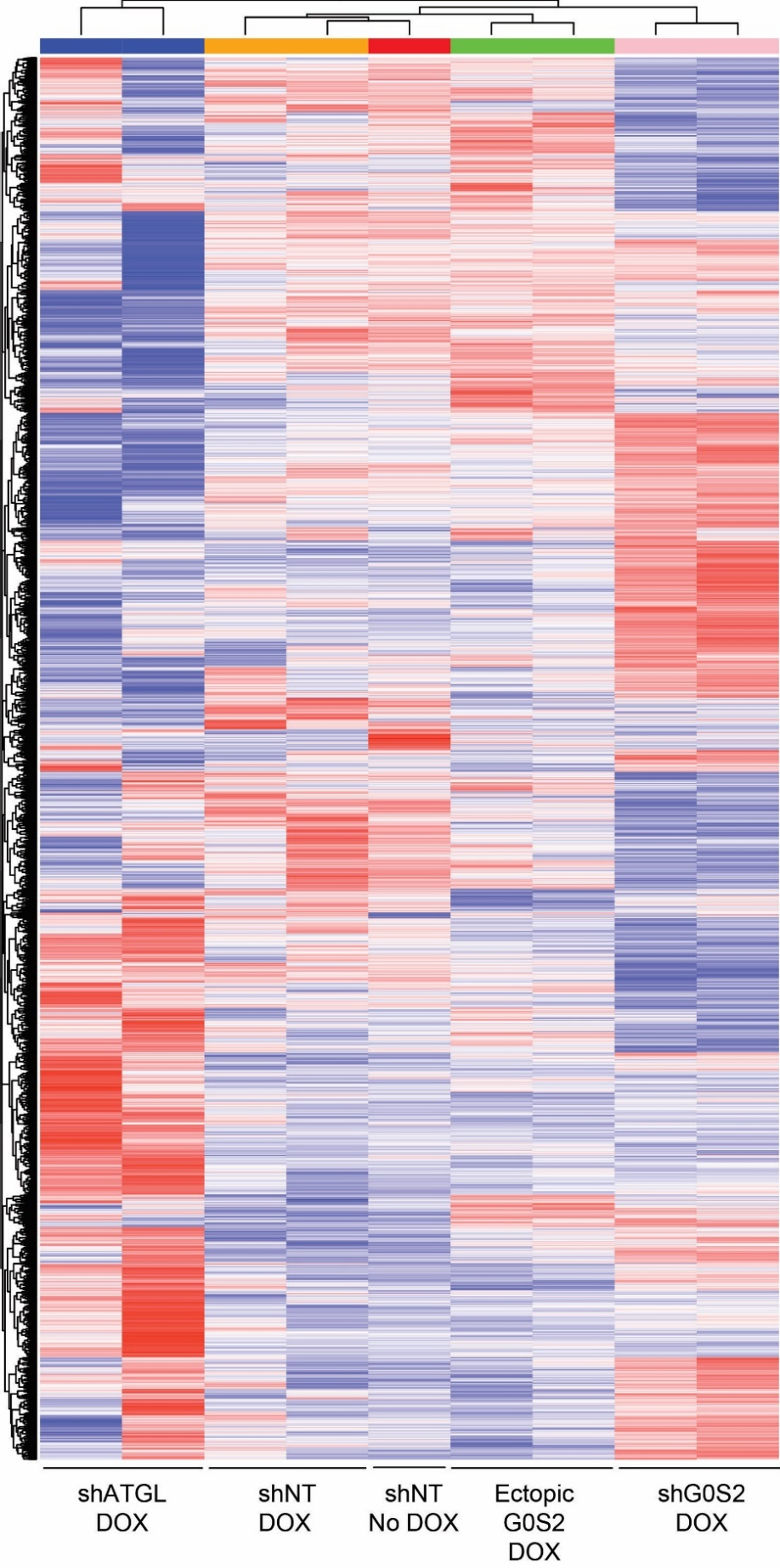


**Figure S8. RNAseq of K562 cells with ectopic G0S2, shG0S2, or shATGL.** The heatmap shows differentially expressed genes comparing K562 cells expressing a non-targeting control shRNA (shNT), pLVX-G0S2 for ectopic expression, or shG0S2 for G0S2 knockdown. All cells were cultured in doxycycline (0.1 μg/ml, 72 h) to induce ectopic expression or knockdown (n=2/group).


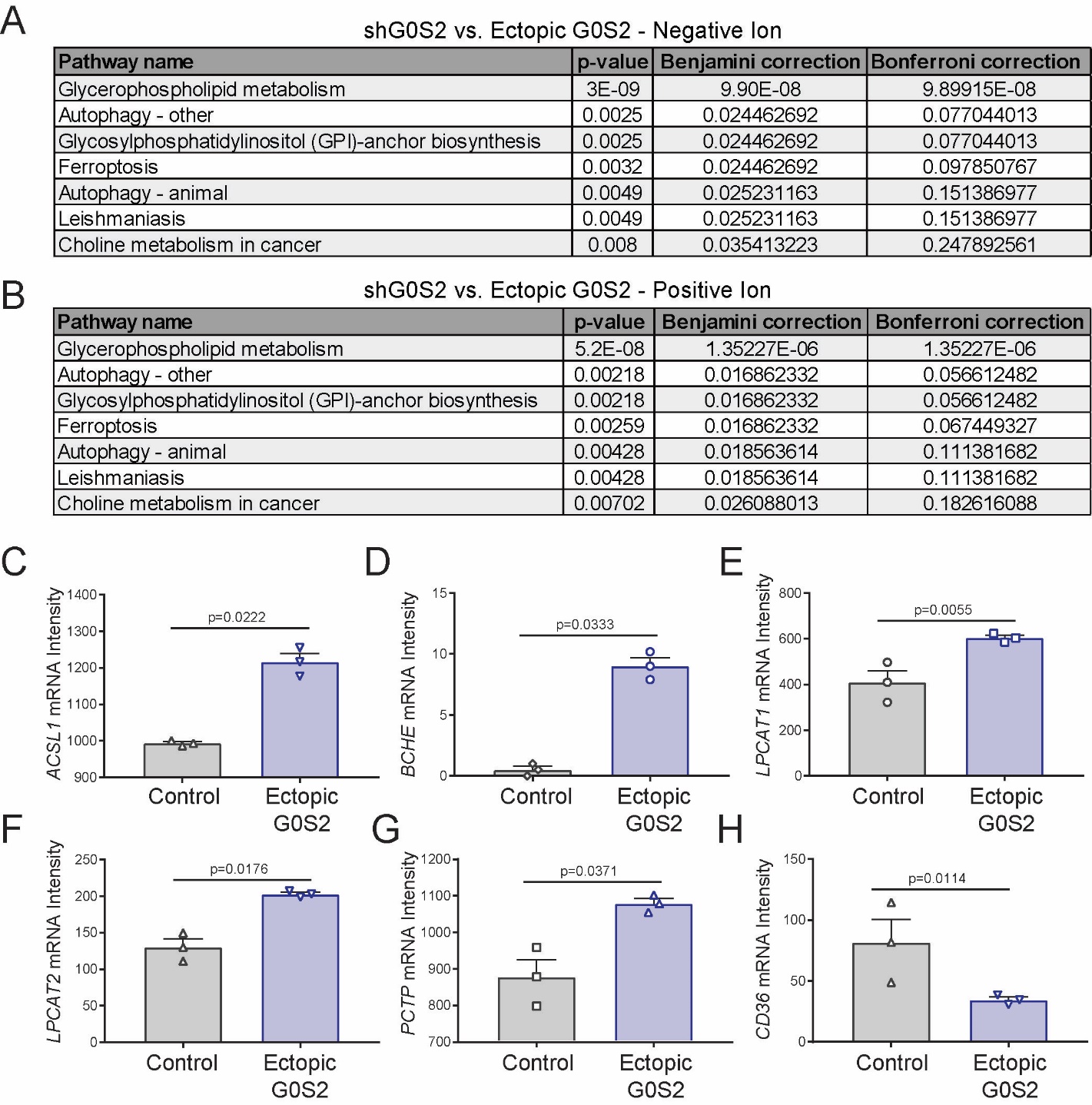


**Figure S9. Ectopic G0S2 expression alters the expression of a number of lipid pathways and lipid modifying enzymes in CML. A-B.** We used a free web-based platform called LIPEA (Lipid Pathway Enrighment Analysis) that can identify the specific pathways that are associated with multiple lipids provided by the user. The tables show the lipid pathways that were enriched by LC/MS-based lipidomics analyses on the negative (**A**) and positive (**B**) ions for K562 cells comparing G0S2 ectopic expression and knockdown (n=6/group). **C-H.** The bar graphs show the mRNA intensity for the genes encoding ACSL1 (**C**), BCHE (**D**), LPCAT1 (**E**), LPCAT2 (**F**), PCTP (**G**), and CD36 (**H**) in K562 cells expressing a control vector or ectopic G0S2 in the presence of doxycycline (0.1 μg/ml, 72h) (n=3/group). ACSL1, Acyl-Co-A synthetase long chain family member 1; BCHE, Butyrylcholinesterase; LPCAT1, Lysophosphatidylcholine acyltransferase 1; LPCAT2, Lysophosphatidylcholine acyltransferase 2; PCTP, Phosphatidylcholine transfer protein; CD36, Cluster of differentiation 36.

## References

1. Eiring AM, Page BD, Kraft IL, et al. Combined STAT3 and BCR-ABL1 inhibition induces synthetic lethality in therapy-resistant chronic myeloid leukemia. *Leukemia*. 2015;29(3):586-97.

2. Chanput W, Mes JJ, Wichers HJ. THP-1 cell line: an in vitro cell model for immune modulation approach. *Int Immunopharmacol*. 2014;23(1):37-45.

3. Tsuchiya S, Kobayashi Y, Goto Y, et al. Induction of maturation in cultured human monocytic leukemia cells by a phorbol diester. *Cancer Res*. 1982;42(4):1530-6.

4. Tsuchiya S, Yamabe M, Yamaguchi Y, Kobayashi Y, Konno T, Tada K. Establishment and characterization of a human acute monocytic leukemia cell line (THP-1). *Int J Cancer*. 1980;26(2):171-6.

5. Barrett T, Wilhite SE, Ledoux P, et al. NCBI GEO: archive for functional genomics data sets--update. *Nucleic Acids Res*. 2013;41(Database issue):D991-5.

6. Edgar R, Domrachev M, Lash AE. Gene Expression Omnibus: NCBI gene expression and hybridization array data repository. *Nucleic Acids Res*. 2002;30(1):207-10.

7. Choi J, Pacheco CM, Mosbergen R, et al. Stemformatics: visualize and download curated stem cell data. *Nucleic Acids Res*. 2018;47(D1):D841-D846.

8. Bagger FO, Kinalis S, Rapin N. BloodSpot: a database of healthy and malignant haematopoiesis updated with purified and single cell mRNA sequencing profiles. *Nucleic Acids Res*. 2019;47(D1):D881-d885.

9. Rhodes DR, Yu J, Shanker K, et al. ONCOMINE: a cancer microarray database and integrated data-mining platform. *Neoplasia*. 2004;6(1):1-6.

10. Seita J, Sahoo D, Rossi DJ, et al. Gene Expression Commons: an open platform for absolute gene expression profiling. *PLoS One*. 2012;7(7):e40321.

11. Zhang X, Ren R. Bcr-Abl efficiently induces a myeloproliferative disease and production of excess interleukin-3 and granulocyte-macrophage colony-stimulating factor in mice: a novel model for chronic myelogenous leukemia. *Blood*. 1998;92(10):3829-40.

12. Kinstrie R, Karamitros D, Goardon N, et al. Heterogeneous leukemia stem cells in myeloid blast phase chronic myeloid leukemia. *Blood Adv*. 2016;1(3):160-169.

13. Yamada T, Park CS, Shen Y, Rabin KR, Lacorazza HD. G0S2 inhibits the proliferation of K562 cells by interacting with nucleolin in the cytosol. *Leuk Res*. 2014;38(2):210-7.

14. Ma T, Lopez-Aguiar AG, Li A, et al. Mice lacking G0S2 are lean and cold-tolerant. *Cancer Bio Ther*. 2014;15(5):643-50.

15. McWeeney SK, Pemberton LC, Loriaux MM, et al. A gene expression signature of CD34+ cells to predict major cytogenetic response in chronic-phase chronic myeloid leukemia patients treated with imatinib. *Blood*. 2010;115(2):315-25.

16. Zheng C, Li L, Haak M, et al. Gene expression profiling of CD34+ cells identifies a molecular signature of chronic myeloid leukemia blast crisis. *Leukemia*. 2006;20(6):1028-34.

17. Diaz-Blanco E, Bruns I, Neumann F, et al. Molecular signature of CD34(+) hematopoietic stem and progenitor cells of patients with CML in chronic phase. *Leukemia*. 2007;21(3):494-504.

18. Neumann F, Teutsch N, Kliszewski S, et al. Gene expression profiling of Philadelphia chromosome (Ph)-negative CD34+ hematopoietic stem and progenitor cells of patients with Ph-positive CML in major molecular remission during therapy with imatinib. *Leukemia*. 2005;19(3):458-60.

19. Berg JS, Lin KK, Sonnet C, et al. Imprinted genes that regulate early mammalian growth are coexpressed in somatic stem cells. *PLoS One*. 2011;6(10):e26410.

20. Chambers SM, Boles NC, Lin KY, et al. Hematopoietic fingerprints: an expression database of stem cells and their progeny. *Cell Stem Cell*. 2007;1(5):578-91.

21. Di Tullio A, Vu Manh TP, Schubert A, Castellano G, Månsson R, Graf T. CCAAT/enhancer binding protein alpha (C/EBP(alpha))-induced transdifferentiation of pre-B cells into macrophages involves no overt retrodifferentiation. *Proc Natl Acad Sci USA*. 2011;108(41):17016-21.

22. Andersson A, Edén P, Olofsson T, Fioretos T. Gene expression signatures in childhood acute leukemias are largely unique and distinct from those of normal tissues and other malignancies. *BMC Med Gen*. 2010;3:6.

23. Hu X, Chung AY, Wu I, et al. Integrated regulation of Toll-like receptor responses by Notch and interferon-gamma pathways. *Immunity*. 2008;29(5):691-703.

24. Majeti R, Becker MW, Tian Q, et al. Dysregulated gene expression networks in human acute myelogenous leukemia stem cells. *Proc Natl Acad Sci USA*. 2009;106(9):3396-401.

25. Novershtern N, Subramanian A, Lawton LN, et al. Densely interconnected transcriptional circuits control cell states in human hematopoiesis. *Cell*. 2011;144(2):296-309.

26. Wildenberg ME, van Helden-Meeuwsen CG, van de Merwe JP, Drexhage HA, Versnel MA. Systemic increase in type I interferon activity in Sjögren's syndrome: a putative role for plasmacytoid dendritic cells. *Eur J Immunol*. 2008;38(7):2024-2033.
